# A National Snapshot of Introductory Chemistry Instructors and Their Instructional Practices

**DOI:** 10.1021/acs.jchemed.4c00040

**Published:** 2024-03-13

**Authors:** Ying Wang, Naneh Apkarian, Melissa H. Dancy, Charles Henderson, Estrella Johnson, Jeffrey R. Raker, Marilyne Stains

**Affiliations:** † Department of Chemistry, University of Virginia, Charlottesville, Virginia 22904-4319, United States; ‡ School of Mathematical and Statistical Sciences, Arizona State University, Tempe, Arizona 85287-1804, United States; § The Evaluation Center, Western Michigan University, Kalamazoo, Michigan 49008-5252, United States; ∥ Department of Physics and Mallinson Institute for Science Education, Western Michigan University, Kalamazoo, Michigan 49008-5252, United States; ⊥ Department of Mathematics, Virginia Polytechnic Institute and State University, Blacksburg, Virginia 24061-0123, United States; # Department of Chemistry, University of South Florida, Tampa, Florida 33620-5250, United States; ∇ Center for the Improvement of Teaching and Research on Undergraduate STEM Education, University of South Florida, Tampa, Florida 33620-5250, United States

**Keywords:** First-Year Undergraduate/General, Collaborative/Cooperative Learning

## Abstract

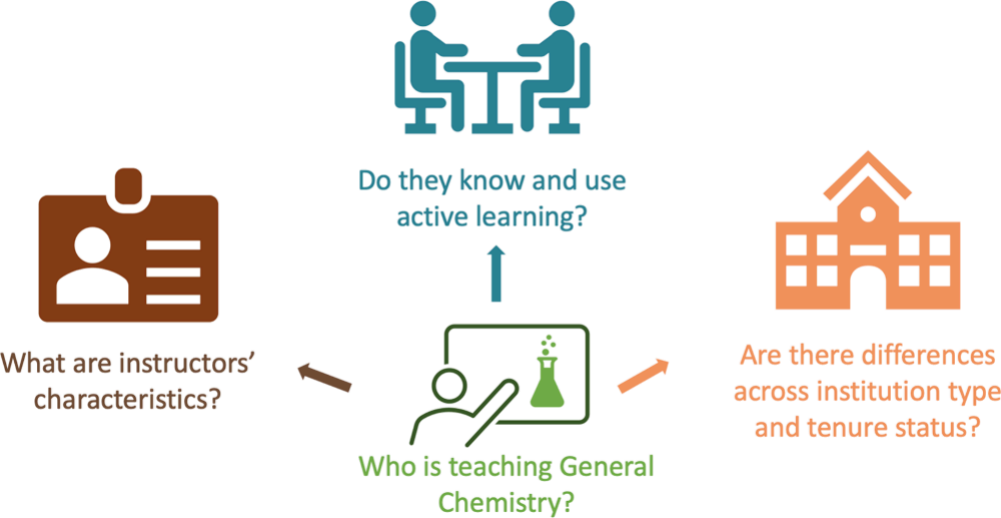

The effectiveness of active learning on promoting students’ academic outcomes and persistence has been established in the literature. However, despite the effort of purposeful change agents, the uptake of active learning in science, technology, engineering, and mathematics (STEM) is slow. While previous research from the chemistry education community has provided insights into the implementation of specific active learning strategies across the United States, the extent to which chemistry instructors leverage these strategies in general remains unknown. This article presents the results of a national survey aimed at exploring introductory chemistry instructors’ knowledge and implementation of active learning, variations on this knowledge, and use across tenure statuses and institution types. This paper also aims to address the gap in the literature in our understanding of the characteristics of instructors of these courses. We thus provide a description of instructors’ demographics, training, teaching experience, and teaching responsibilities. Our findings reveal that instructors in these courses are prominently males of European descent. Additionally, instructors come into their teaching position with minimal pedagogical training and participate mainly in short training once in their position. While the majority of instructors have knowledge of specific active learning strategies, their consistent implementation remains limited, with lecturing still being the instructional practice of choice. Variations were found between institution types and across tenure statuses within institutions in terms of pedagogical training, use of specific active learning strategies, and proportion of class time spent lecturing. The findings provide a baseline for future studies that aim to assess the effectiveness of interventions fostering the implementation of active learning in introductory chemistry courses and highlight the critical need for improved communication about teaching practices across institutions and tenure statuses.

## Introduction

Pedagogies enacted by instructors are key contributors to students’ attrition in science, technology, engineering, and mathematics (STEM) courses.^1,2^ In the late 1990s, Seymour and Hewitt^1^ conducted a study exploring students’ persistence in undergraduate STEM education; they found that 90% of the students who switched out of STEM majors and 74% of those who persisted in STEM majors identified “poor quality STEM teaching” as the most common concern. Seymour, Hewitt, and others replicated their findings 20 years later, where nearly all switchers (96%) and 72% of persisters still reported instructors’ pedagogies and the curriculum enacted as key concerns.^2^ The retention of STEM majors has been linked specifically to students’ experiences in introductory level courses, such as general chemistry, which often function as foundational courses for many STEM majors.^3,4^ For example, one study found that students’ experiences in general chemistry were the principal cause of declined interest in continuing premedical studies and, ultimately, interest in a medical career.^5^ These findings highlight a need for transforming students’ learning experiences in postsecondary level STEM courses, particularly in introductory courses, to improve student outcomes and retention.

### Enactment of Active Learning in STEM Courses

One core suggestion to improve students’ experiences is to shift class periods toward more active learning.^6^ In this study, we operationalize active learning as “the instructional practices that promote students’ engagement in the learning process to facilitate their construction and use of knowledge.”^7^ There has been much development, research, and dissemination of active learning strategies, which has led to substantial evidence for their effectiveness in improving students’ academic performance, attitude, and persistence.^8−11^ Specifically, active learning was shown to be associated with increased conceptual understanding and retention compared to traditional lecture-based instruction,^9^ and it was shown to reduce the opportunity gap between students of European descent and minoritized and historically excluded students.^10^ For example, Rahman and Lewis’^12^ meta-analysis of 99 studies on active learning strategies in chemistry courses, such as process-oriented guided inquiry learning (POGIL), peer-led team learning (PLTL), problem-based learning (PBL), and flipped instruction, found an overall positive association between the implementation of these active learning strategies and students’ academic performance.

Despite the need to transform teaching and the established benefits of active learning, previous research has shown that STEM instructors’ uptake of innovative instructional strategies in in-person settings is slow. Over a decade ago, national survey studies in STEM courses, including physics, engineering, and geosciences, shed light on the knowledge and adoption of specific sets of active learning strategies by STEM instructors across different institutions.^13−15^ These studies showed that although most STEM instructors are familiar with various active learning strategies, only half of them implement one or more of these strategies in their courses. More recently, a large-scale observation-based study indicated that although STEM instructors employed varied teaching practices within their courses, lecturing was still prominent, accounting for 75% of class time.^16^

Of note, the literature has explored the uptake of active learning in terms of two indices: the proportion of class time spent lecturing^16,17^ and the level of use of research-based instructional strategies (RBIS), which are active learning strategies that have strong educational research foundations and evidence.^14,15,18^ We recognize that these two indices have their limitations. For example, the focus on the time spent lecturing during class may lead to the assumption that lecturing is incompatible with active learning and should thus be eliminated. The focus on RBIS creates an assumption that these are the only strategies that support active learning. Neither of these assumptions are accurate. Active learning can take many forms and relies on a deliberate selection of various instructional approaches that may include lecturing and RBIS, with the goal of promoting student engagement in the learning process and construction of knowledge.^7^ While we recognize these limitations, we chose to follow the standards for large-scale studies that are currently established in the literature to provide a means for comparison across studies and settings.

### Enactment of Active Learning in Chemistry Courses

Only a few studies have explored the extent of the implementation of active learning in chemistry courses nationwide. National surveys of postsecondary chemistry instructors were conducted to estimate the use of several popular RBIS, including PLTL, PBL, POGIL, and flipped classrooms.^19,20^ It was found that a quarter or less of the surveyed instructors implemented each of these RBIS. It is worth mentioning that instructors who are not on the tenure track tend to implement PLTL and POGIL more than tenured/tenure-track instructors.^20^ While this work provides valuable insights into the prevalence of these specific strategies, further research is necessary to understand the broader landscape of active learning adoption in chemistry settings across the United States. Our research herein aims to capture the current state of active learning implementation in introductory chemistry courses, serving as a baseline for future studies evaluating the effectiveness of pedagogical reform initiatives in these learning environments.

### Focus on Instructors’ Characteristics

The teacher-centered systemic reform (TCSR) model^21,22^ is a framework that has been extensively leveraged in the literature to explore drivers and barriers to the adoption of active learning.^17,18,20,23,24^ This model, which was developed to examine how reform initiatives influence instructional practices, includes contextual factors (e.g., institution type, tenure status), personal factors (e.g., instructors’ demographic profile, years of teaching experience, teaching-related professional development), and teacher thinking factors (e.g., instructors’ knowledge and beliefs about teaching and learning).^21^ Previous empirical studies^17,18^ explored the associations between these factors with both reduced lecturing time and increased RBIS use.^17,18,24^ Although these studies provide valuable insights in terms of what factors might influence the enactment of active learning, little is known about the descriptive aspects of these factors. Thus, in this work, along with providing a snapshot of the level of awareness and use of active learning practices in introductory chemistry courses, we also aim to provide a description of the instructors who students are likely to learn from in introductory chemistry courses in terms of their demographics, training, teaching experience, and teaching responsibilities. This information is not readily available to the chemistry education community but can be extremely useful to understand the need for the diversification and training of the introductory chemistry instructor workforce. Moreover, we describe the variations in knowledge and use of active learning across different academic appointments the instructors hold (i.e., tenure status) and their academic environment (i.e., institution type). Indeed, prior studies have provided contradictory results regarding the relationship between tenure status and the implementation of active learning practices. For example, some studies have reported a relationship between tenure status and the adoption of RBIS,^20,23,25^ while others have not found such a relationship.^18^ This information is critical to inform the implementation of more chemistry- and context-specific instructional reform efforts, which are more likely to be successful than broad stroke approaches.

## Research Questions

The overarching goal of the work reported herein is to provide insights into the current state of active learning implementation in introductory chemistry courses and identify key opportunities for future research and reform efforts that are tailored to the specific needs of different chemistry instructor populations. To address these questions, we will address the following research questions:

1. What are the demographics, training, and teaching responsibilities of introductory chemistry instructors in the United States?
2. To what extent do introductory chemistry instructors implement active learning?

These research questions were examined across four institution types per the Carnegie Basic Classification of Institutions of Higher Education: associate degree-granting institutions (referred to as A.A. institutions hereafter), bachelor’s degree-granting institutions (referred to as B.A./B.S. institutions hereafter), master’s degree-granting institutions (referred to as M.A./M.S. institutions hereafter), and doctoral degree-granting institutions (referred to as Ph.D. institutions hereafter). All research questions were further explored by comparing instructors across different tenures within each institution.

## Methods

This study employs data from a large, collaborative project that aimed to explore factors associated with postsecondary instructors’ usage of active learning in introductory chemistry, mathematics, and physics courses.^17,18,26,27^ Data were collected through a national survey designed to target five key topics: (1) context and details; (2) instructional practice; (3) awareness and usage of active learning; (4) perceptions, beliefs, and attitudes related to students, learning, and departmental context; and (5) personal demographics and experience. Details regarding data collection and survey development are reported elsewhere.^17,18^ Herein, we present an overview of the survey questions analyzed in this study and the survey administration procedures used for the overall project.

### Survey Questions

A full list of the survey questions that were employed in this study is provided in the Supporting Information. We present here an overview of some specific questions to help readers better conceptualize the study results. Respondents were asked to indicate the level of professional development (PD) experiences that they received prior to their teaching appointment by providing the number of teaching-focused academic courses that they completed at the undergraduate, graduate, and postdoctoral levels. Response options were 0, 1, 2, 3, or 4+, and respondents selected a single option from the provided drop-down menu; 4+ was tabulated as 4 for the purpose of statistical analyses. Additionally, respondents were asked about their participation in various types of teaching-related PD programs since their appointment as an instructor at any institution. They were asked to indicate whether they participated in one or more of the following options: half-day workshop(s), full-day or longer workshop(s), attending a teaching-focused conference, regular meetings as part of a formal program, new faculty experience at my institution, and a new faculty workshop external to my institution. A total score for each participant was calculated based on the number of PD types they participated in.

The level of use of active learning was measured in two different ways, following the standards in the literature. First, the knowledge and use of RBIS were probed using two different questions. In the first question, respondents were asked about whether they are aware of and use RBIS using a Guttman scale.^28^ The scale contains a list of six statements related to this question, and respondents have to provide a yes/no response to each statement (see question 18 of the survey in the Supporting Information). Following this question, a list of specific RBIS with definitions was presented to the instructors, and the instructors were asked to rate their level of familiarity with and usage of each RBIS on a scale from 1 to 5 (1 = “I have never heard of this”; 2 = “I know the name, but not much more”; 3 = “I know about this, but have never used it in my course”; 4 = “I have tried it in this course, but no longer use it”; and 5 = “I currently use it in this course to some extent”). Second, instructors were asked to indicate the proportion of time during regular class meetings that students engage in four different activities: working individually, working in small groups, participating in whole-class discussions, and listening to the instructor lecture or solve problems, a proxy for percent time lecturing.

### Survey Administration

The survey was administered online in the spring of 2019. A database consisting of 18,337 postsecondary instructors teaching introductory chemistry, mathematics, and physics courses in the United States was constructed by the authors, and respondents were identified from this database. With the aim of constructing a representative sample from different types of degree-granting institutions, including A.A., B.A./B.S., M.A./M.S., and Ph.D. institutions, a stratified census sampling strategy was employed. To get the contact information for potential participants, the authors contacted department chairs at selected institutions using the instructors’ contact information that was compiled by the American Institute of Physics Statistical Research Center.

Western Michigan University Human Subjects Institutional Review Board approved the project under the exempt review category (Application No. 17-06-10). Consent was obtained from the survey participants electronically.

The study was conducted before the pandemic and aimed to explore instructors’ pedagogy in in-person courses, which was the dominant form of course delivery at the time. Thus, data were cleaned by including only those who met specific criteria: those who (1) taught introductory chemistry courses during the 2017–18 or 2018–19 academic years for the main whole-class meetings of the course, (2) facilitated courses that were not entirely taught online, (3) provided consent to participate in the study, and (4) responded to at least one question for the current study. The final sample for our work includes 1232 chemistry instructors. Our sample comprises instructors from four types of institutions: A.A. institutions (33.8%), B.A./B.S. institutions (30.6%), M.A./M.S. institutions (13.6%), and Ph.D. institutions (22.0%). Sample sizes vary for each statistical test.

## Results

### Introductory Chemistry Instructors’ Demographics

Table 1 provides the demographics of the introductory chemistry instructors in this sample. Across institutions, students are more likely to have a man than a woman as an instructor (Table 2). Moreover, the instructor is very likely to be of European descent, which is often described as White in the United States (Table 1). Notably, the proportion of instructors of European descent differs across institutions [χ^2^(1,1018) = 28.633, *p* < 0.001, *V* = 0.168]. Students at B.A./B.S. institutions are much more likely than students at other institutions to be taught by a person of European descent (Table 3).

**Table 1:** Introductory Chemistry Instructors’ Demographics

demographic type	label	percentage of respondents (%)
gender (*n* = 1067)	men	56.0
	women	42.2
	transgender	0.1
	genderfluid	0.1
	prefer not to answer	1.7
race and ethnicity (*n* = 1062)	European	79.1
	Hispanic and/or Latinx	5.1
	East Asian	4.1
	Black and/or African American	3.8
	Middle Eastern and/or North African	2.5
	South Asian	2.4
	Southeast Asian	2.3
	American Indian and/or Alaska Native	1.1
	Central Asian	0.3
	Native Hawaiian and/or Pacific Islander	0.1
	not listed	0.1
	prefer not to answer	4.1

**Table 2:** Introductory Chemistry Instructors’ Gender by Institution Type^a^

	percentage of total participants in each institution type (%)			
	A.A. (*n* = 329)	B.A./B.S. (*n* = 334)	M.A./M.S. (*n* = 147)	Ph.D. (*n* = 238)
men	53.8	56.0	56.5	63.4
women	46.2	44.0	43.5	36.6

a Instructors who preferred not to answer the questions were excluded from this table.

**Table 3:** Introductory Chemistry Instructors’ Race/Ethnicity by Institution Type^a^

	percentage of total participants in each institution type (%)			
	A.A. (*n* = 317)	B.A./B.S. (*n* = 328)	M.A./M.S. (*n* = 139)	Ph.D. (*n* = 234)
European	77.9	90.1	73.4	82.9
non-European	22.1	9.1	26.6	17.1

a Instructors who preferred not to answer the question were excluded from this table.

The majority of the participating instructors teach at institutions with a tenure system; institutions lacking a tenure system are typically A.A. institutions (Table 5). Students are taught by faculty of different ranks, including full professors (32%), associate professors (20%), and assistant professors (18%) (Table 4). The distribution of instructors by tenure status varies across the institutions [χ^2^ (9,1023) = 189.741, *p* < 0.001, *V* = 0.249]. Students are more likely to have an introductory chemistry instructor on the tenure-track system at B.A./B.S. institutions than at any other institutions. On the other end, M.A./M.S. and Ph.D. institutions rely more on non-tenure-track instructors to teach introductory chemistry than other institutions (Table 5). Details of instructors’ gender and race/ethnicity by tenure status by institution type are available in the Supporting Information (Tables S1 and S2).

**Table 4:** Instructors’ Academic Rank and Tenure Status

statistical category	label	percentage of total participants (%)
academic rank (*n* = 1079)	professor	32.2
	lecturer/instructor	25.4
	associate professor	19.9
	assistant professor	18.1
	visiting professor/lecturer/instructor	3.7
	graduate student instructor or teaching assistant	0.5
	postdoctoral instructor	0.3
tenure status (*n* = 1023)	tenured	51.3
	on tenure track, but not tenured	16.5
	not on a tenure track, but this institution has a tenure system	23.9
	no tenure system at this institution	8.2

**Table 5:** Instructors’ Tenure Status by Institution Type

	percentage of total participants in each institution type (%)			
tenure status	A.A. (*n* = 323)	B.A./B.S. (*n* = 323)	M.A./M.S. (*n* = 147)	Ph.D. (*n* = 230)
tenured	44.0	65.0	42.2	48.3
on tenure track	13.3	19.8	25.2	10.9
not on a tenure track	20.7	12.4	32.0	39.6
no tenure system	22.0	2.8	0.7	1.3

### Introductory Chemistry Instructors’ Training

Students are taught by instructors with extensive training in chemistry, with over 90% of instructors at B.A./B.S., M.A./M.S., and Ph.D. institutions and 66% of instructors at A.A. institutions holding a doctoral degree (Table S3). Instructors also have extensive teaching experience, with more than half of the instructors at each institution type having 10 or more years of teaching experience (Table S4).

We captured introductory chemistry instructors’ pedagogical training by tabulating their engagement in teaching-related professional development (PD) experiences. In particular, we asked instructors to report on (1) the number of teaching-related courses they took prior to beginning their teaching appointment and (2) the different types of teaching-focused PD programs they attended after they started their teaching appointment.

**Figure 1:**
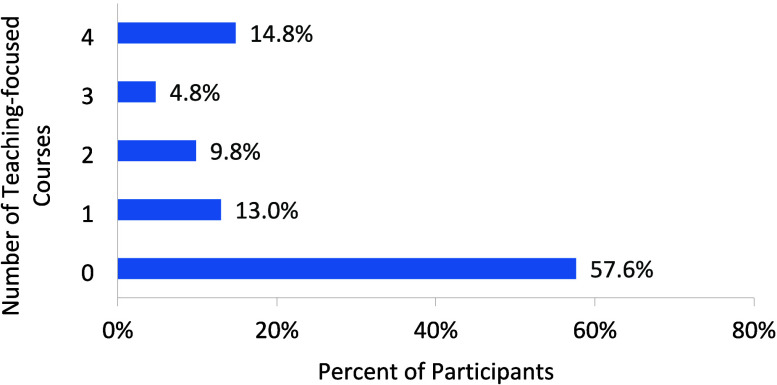
Number of teaching-focused courses that introductory chemistry instructors experienced prior to starting their academic career (i.e., as an undergraduate, graduate student, or postdoctoral scholar).

Over two-thirds of the instructors had minimal or no PD experience prior to starting their teaching appointment (Figure 1). Specifically, the average number of teaching-focused courses completed is 1.1, with 58% of instructors reporting that they had never completed any teaching-focused coursework at the undergraduate, graduate, and postdoctoral levels prior to their first appointment as an instructor.

We explored the variation of instructors’ prior PD experience across institution types by conducting a one-way analysis of variance (ANOVA) on the average number of teaching-focused courses. The results indicate a statistically significant difference across institution types [*F*(3, 925) = 14.62, *p* < 0.001, η^2^ = 0.045] (see Table 6). Tukey-HSD (honestly significant difference) pairwise comparisons revealed that A.A. instructors had completed significantly more courses than both B.A./B.S. instructors (*p* < 0.001, Cohen’s *d* = 0.74) and Ph.D. instructors (*p* < 0.001, Cohen’s *d* = 0.55).

**Table 6:** Comparison on PD Experience prior to Starting a Teaching Appointment across Institution Types

	mean						
	A.A.	B.A./B.S.	M.A./M.S.	Ph.D.	*F*-value	*p*-value	η^2^
teaching-focused courses^a^	1.5 ± 0.1	0.7 ± 0.1	1.1 ± 0.1	0.9 ± 0.1	14.62	<0.001	0.045
PD types	3.6 ± 0.1	3.6 ± 0.1	3.5 ± 0.1	3.2 ± 0.1	4.466	0.004	0.013

a Rating on courses taken prior to teaching career was on a scale of 1, 2, 3, 4+, and 4+ was treated as 4 in the analysis.

Furthermore, a series of one-way ANOVAs was performed to explore the variation in instructors’ PD experiences prior to their academic appointment across tenure statuses within each institution. Significant differences were observed for the following three institution types: B.A./B.S. [*F*(2, 272) = 5.149, *p* = 0.006, η^2^ = 0.103], M.A./M.S. [*F*(2, 126) = 11.331, *p* = 0.003, η^2^ = 0.089] and Ph.D. [*F*(2, 201) = 8.689, *p* < 0.001, η^2^ = 0.082] (see Table 7). Results of Tukey-HSD pairwise comparisons suggest that at Ph.D. institutions, non-tenure-track instructors have significantly more professional development experiences prior to starting their teaching appointment compared to their tenured counterparts (*p* < 0.001, Cohen’s *d* = 0.87) after a Bonferroni correction.

**Table 7:** Comparison of Number of Teaching-Related Courses Taken prior to Starting a Teaching Appointment by Tenure Status within Each of the Four Types of Institutions

	mean						
type of institution	tenured	on tenure track	not on tenure track	no tenure system	*F*-value	*p*-value	η^2^
A.A.	1.5 ± 0.1	1.8 ± 0.3	1.2 ± 0.2	1.6 ± 0.2	0.855	0.465	0.01
B.A./B.S.	0.5 ± 0.1	1.0 ± 0.2	1.2 ± 0.3	-	5.149	0.006	0.036
M.A./M.S.	0.6 ± 0.2	1.5 ± 0.3	1.5 ± 0.3	-	5.972	0.003	0.089
Ph.D.	0.5 ± 0.1	1.0 ± 0.3	1.3 ± 0.2	-	8.689	<0.001	0.082

Once instructors started their appointment, they engaged in or took advantages of more training opportunities and participated in an average of 3.0 different types of teaching-focused PD experiences (Figure S1). The most popular types of PD were full-day (81.8%) and half-day workshops (75.0%) (Figure S2). An exploration of the variations of post-appointment PD across institution types shows a statistically significant difference [*F*(3, 1016) = 4.466, *p* = 0.004, η^2^= 0.013]. Tukey-HSD pairwise comparisons revealed that A.A. instructors attended significantly more types of PD after they started their teaching appointment than Ph.D. instructors (*p* = 0.006, Cohen’s *d* = 0.57). With respect to the tenure status within each institution (Table 8), significant differences were observed for two institutions: A.A. [*F*(3, 207) = 11.331, *p* < 0.001, η^2^ = 0.103] and Ph.D. [*F*(2, 213) = 5.91, *p* = 0.003, η^2^ = 0.053]. Results of Tukey-HSD pairwise comparisons suggest that at Ph.D. institutions, non-tenure-track instructors have significantly more PD experiences compared to tenured instructors after starting their teaching appointment (*p* < 0.001, Cohen’s *d* = 0.79). In contrast, at A.A. institutions, non-tenure-track instructors reported significantly fewer types of PD after starting their teaching appointment than tenured (*p* < 0.001, Cohen’s *d* = 1.25) and tenure-track instructors (*p* < 0.001, Cohen’s *d* = 1.14; see Tables 8).

**Table 8:** Comparison of Number of Teaching-Related PD Types after Starting a Teaching Appointment by Tenure Status within Each of the Four Types of Institutions

	mean						
type of institution	tenured	on tenure track	not on tenure track	no tenure system	*F*-value	*p*-value	η^2^
A.A.	4.0 ± 0.1	4.0 ± 0.2	2.8 ± 0.2	3.4 ± 0.2	11.331	<0.001	0.103
B.A./B.S.	3.8 ± 0.1	3.3 ± 0.2	3.3 ± 0.2	-	3.297	0.038	0.022
M.A./M.S.	3.7 ± 0.2	3.4 ± 0.2	3.3 ± 0.3	-	1.095	0.337	0.015
Ph.D.	2.8 ± 0.2	3.3 ± 0.3	3.6 ± 0.2	-	5.911	0.003	0.053

### Instructors’ Teaching Load

#### Course Enrollment and Number of Courses Taught per Semester

We gathered data from instructors regarding their course enrollment and the number of courses that they teach each semester. The findings illustrate notable differences among instructors at different types of institution. Specifically, A.A., B.A./B.S., and M.A./M.S. instructors reported teaching approximately three courses per semester (3.1 courses on average; see Table S5) with an enrollment average in each section ranging from 30.4 students for A.A. instructors to 79.1 students for M.A./M.S. instructors (Table S6). In contrast, Ph.D. instructors taught fewer courses per semester (1.9 courses on average), but their course enrollment was substantially higher, with an average of 193.9 students per section of a course.

#### Course Design Decisions

**Figure 2:**
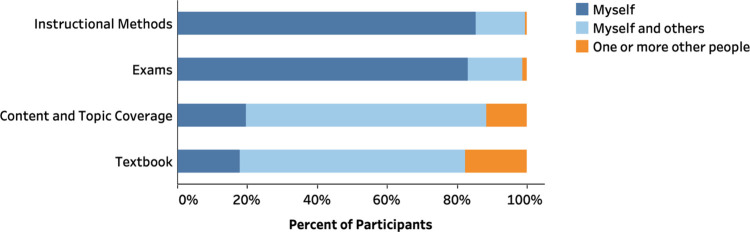
Distributions of decision makers on content and topic coverage, textbook, exams, and instructional methods.

We explored instructors’ level of autonomy in teaching by asking them to identify the decision makers on four course components: instructional methods, exams, content and topic coverage, and textbook. While respondents tended to share decision-making for textbook and course content, they generally exercised independent decision-making for exams and instructional methods, indicating a high level of autonomy for these two components (Figure 2 and Table S7). The distribution of decision makers varied across different types of institutions for textbook [χ^2^(6,1220) = 90.538, *p* < 0.001, *V* = 0.193], content coverage [χ^2^(6,1222) = 71.549, *p* < 0.001, *V* = 0.171], instructional methods [χ^2^(6,1227) = 28.061, *p* < 0.001, *V* = 0.107], and exams [χ^2^(6,1227) = 68.235, *p* < 0.001, *V* = 0.167]. A.A. instructors are more likely to make their own decisions about curricular components (i.e., textbook and content coverage), even though less than 30% of A.A. instructors do so (Tables S10 and S11). Ph.D. instructors are more likely to work with others on the design of exams and implementation of the instructional method, keeping in mind that only 29% and 20% work with others on exams and instructional methods, respectively (Tables S8 and S9).

### RBIS Knowledge and Usage

**Figure 3:**
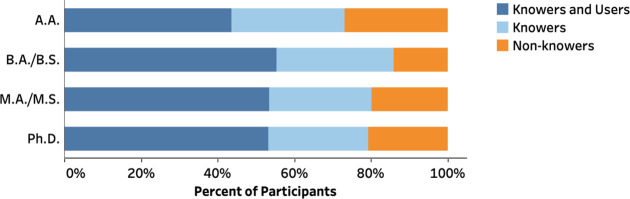
Distribution of instructors in terms of their knowledge and usage of RBIS.

Instructors were asked to report on their knowledge and use of RBIS. Overall, 80% of the respondents are aware of RBIS, but only 51% consistently use RBIS in their classes. Chi-square tests indicate a significant difference across institution types in the distribution of instructors who knew RBIS (referred to as “Knowers”) [χ^2^(3,1145) = 18.063, *p* < 0.001, *V* = 0.126] and the distribution of instructors who knew and consistently used RBIS (referred to as “Knowers and Users”) [χ^2^(6,1145) = 12.014, *p* = 0.007, *V* = 0.102]. A.A. instructors are less likely to report awareness of RBIS with 73% indicating awareness of RBIS, in contrast to 86% of B.A./B.S. respondents. Among instructors who reported being aware of RBIS, respondents from A.A. institutions had the lowest proportion of consistent users (see Figure 3 and Table S12).

**Figure 4:**
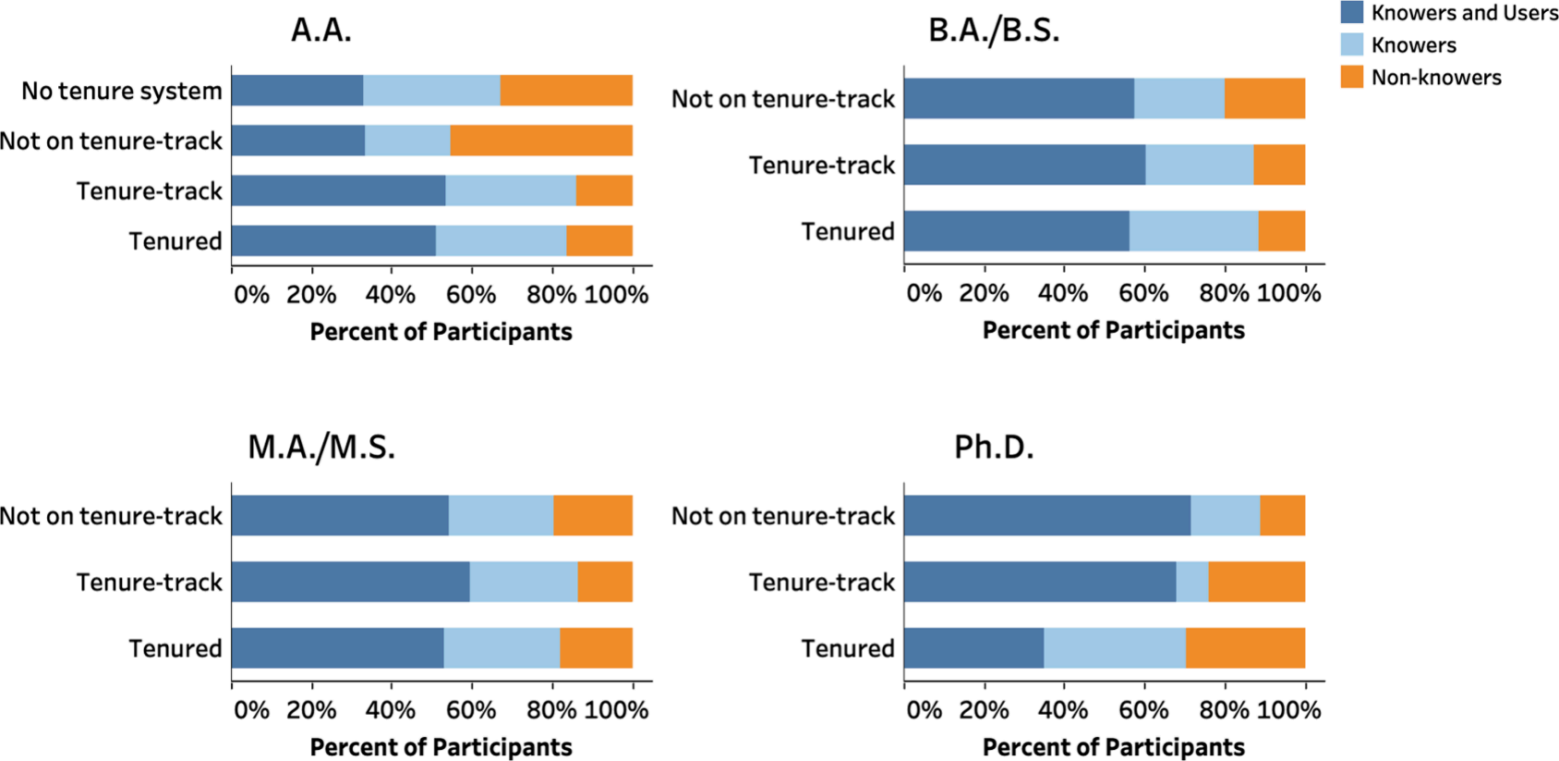
Distribution of instructors in terms of their knowledge and usage of RBIS by tenure status within institution type.

Knowledge and usage of RBIS were further explored within each institution type (Figure 4 and Tables S13–S16). A striking variation between tenure statuses was observed for A.A. and Ph.D. institutions. At A.A. institutions, only 45% of the non-tenure-track instructors knew about RBIS, in contrast to over 79% of tenure-track and tenured instructors at this type of institution. Furthermore, at Ph.D. institutions, 68% of tenured instructors reported to know of RBIS, but only 48% of this group consistently used RBIS. Furthermore, 88% of the non-tenure-track instructors at Ph.D. institutions reported to know of RBIS with 73% of this group consistently using RBIS. Note that the sample size of tenure-track instructors at Ph.D. institutions is relatively small (*n* = 25); therefore, we are cautious about making too many broad claims about this group.

**Figure 5:**
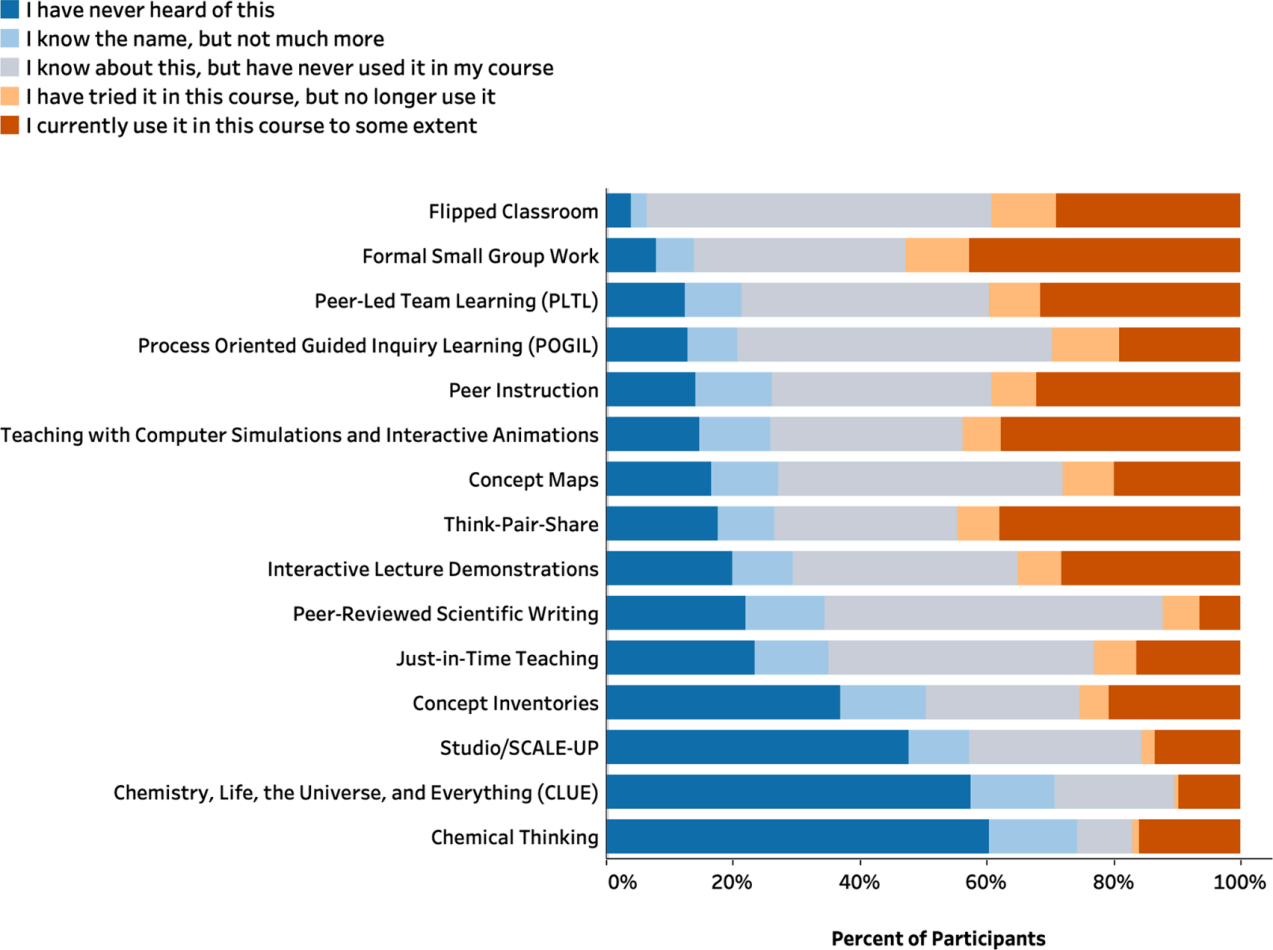
Distribution of instructors in terms of their knowledge and usage of specific RBIS.

In addition to general knowledge and use of RBIS, we also explored respondents’ awareness and usage of specific RBIS (Figure 5 and Table S17). The three most-consistently used RBIS, indicated by the proportion of instructors who rated “I currently use it in this course to some extent”, are formal small group work, teaching with computer simulations and interactive animations, and think-pair-share. Around half of the instructors indicated that they had never heard of Chemical Thinking, Chemistry, Life, the Universe, and Everything (CLUE) or Studio/SCALE-UP. For many RBIS, more than one-third of the instructors indicated that they knew about the RBIS but never used it in their course.

### Distribution of Class Time

Instructors were asked to estimate the proportion of class time that they spent on four different activities. They reported spending the majority of their class time lecturing (58.5% of class time), but they also employed some small group work (18.3%), individual work (18.3%), and whole-class discussions (10.9%).

**Figure 6:**
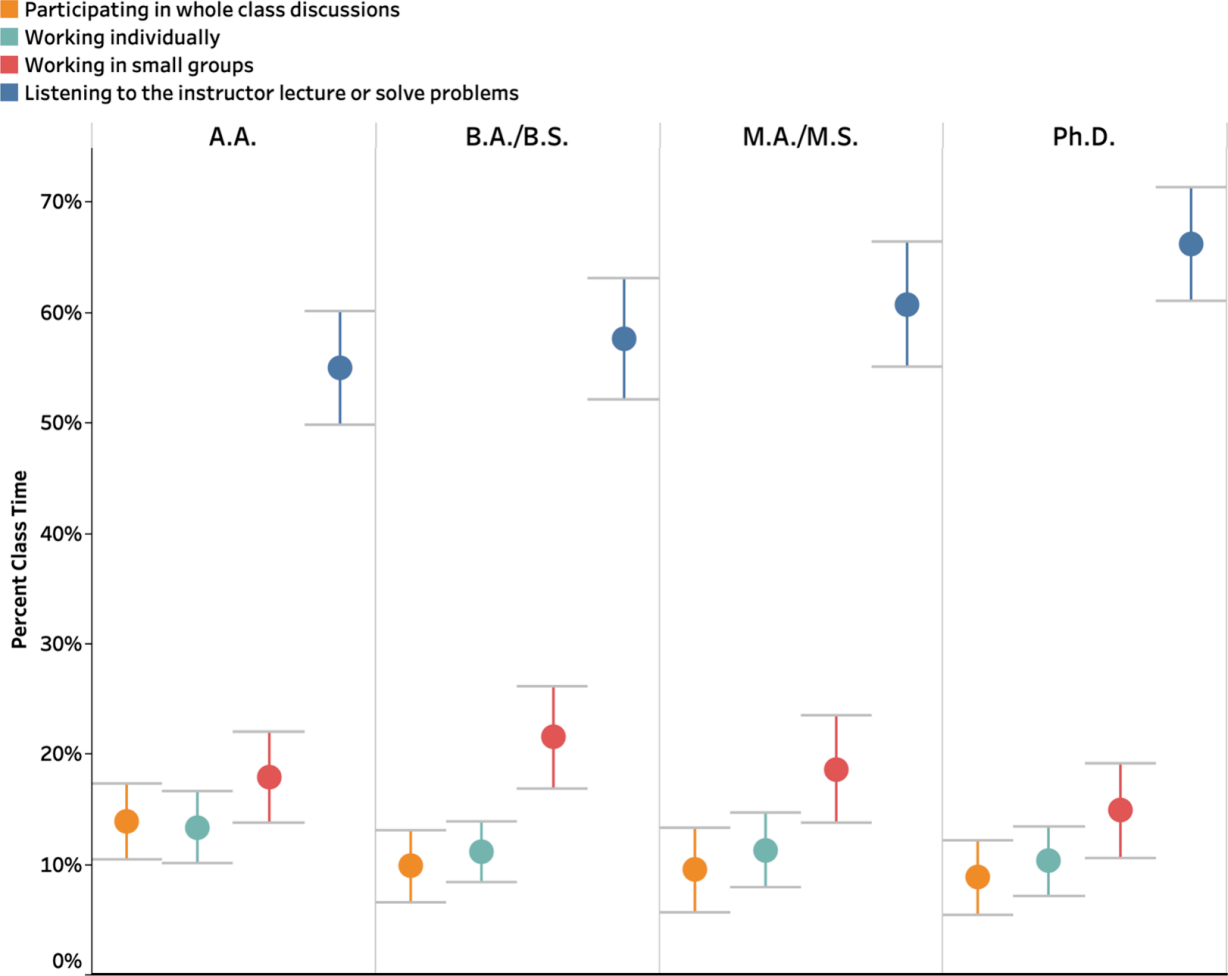
Percent class time allocated to four class activities across institution types.

Variations in class time distribution across different types of institutions were explored (Figure 6 and Table S18). Instructors at Ph.D. institutions spent more time lecturing (66%) and less time on other activities compared to their counterparts at other institutions. A.A. instructors spent less time lecturing (55%) and more time on individual work (13%) or whole-class discussions (14%), while instructors at B.A./B.S. institutions spent a greater proportion of class time on small group work (22%) compared to instructors at other institution types. The results of a one-way ANOVA revealed a statistically significant difference in lecturing time across institutions [*F*(3, 1209) = 11.930, *p* < 0.001, η^2^ = 0.03]. Tukey-HSD pairwise comparisons suggest that the significant differences in lecturing time were between instructors at Ph.D. and A.A. institutions (*p* < 0.001, Cohen’s *d* = 0.67), as well as between instructors at Ph.D. and B.A./B.S. institutions (*p* < 0.001, Cohen’s *d* = 0.50). Furthermore, multiple one-way ANOVAs were conducted to explore the lecturing time variation across different tenure statuses within each institution type (Table S19). A statistically significant difference was found only at Ph.D. institutions [*F*(2, 226) = 7.42, *p* < 0.001, η^2^ = 0.06]. Tukey-HSD comparisons revealed that tenured instructors at Ph.D. institutions spent significantly more time lecturing than non-tenure-track instructors (*p* = 0.002, Cohen’s *d* = 0.67).

## Discussion and Implications

Our study aimed to describe the current state of active learning implementation in introductory chemistry courses and identify key opportunities for future research and reform efforts that are tailored to the specific needs of different chemistry instructor populations. Particularly, we sought to capture the nuances across institution types and tenure statuses regarding the above-mentioned aspects. To achieve these goals, we gathered data from a group of introductory chemistry instructors (*n* = 1232) across the United States using survey research methods. The findings and implications for departmental and instructional leaders, individual faculty members, education researchers, and other professionals seeking to promote active learning adoption in postsecondary introductory courses are discussed in the following sections.

### Lack of Diversity among Introductory Chemistry Instructors

The analysis of instructors’ demographics indicates that the majority of the instructors (79%) are of European descent, while Black instructors and Hispanic instructors account for only 4% and 5% of the sample, respectively. Furthermore, our sample consisted of fewer female instructors (42%) than male instructors (56%). When compared to the United States census data, which shows that the population contains 50.5% female persons, 19.1% Hispanic persons, and 13.6% Black persons, the underrepresentation of Black, Hispanic, and female instructors in our sample becomes apparent. Research suggests that female instructors and instructors of racial minorities can act as role models and inspire minority students to pursue their academic goals.^29,30^ Numerous empirical studies in higher education have shown that the presence of instructors from diverse racial and gender backgrounds positively impacts the academic performance and career aspirations of students who share the same race and gender identities.^30−33^ Having more instructors from diverse racial and gender groups is, therefore, beneficial to making chemistry learning environments more equitable. However, considering the long-standing issue of the racial and gender gaps in the STEM workforce,^34^ institutions may face the difficult task of recruiting instructors from these groups. This creates a cycle that perpetuates the underrepresentation of minorities in both the student body and the instructor body. Therefore, it is essential to break this cycle by taking steps to increase diversity among students and support underrepresented minority students and female students in their pursuit of chemistry careers. In alignment with the primary goal of this paper, it is important to note that implementing active learning strategies such as RBIS has been shown to be one effective way to narrow the opportunity gap between minoritized students and their counterparts.^10,35^ Thus, facilitating instructors to adopt more active learning in their classrooms can be an effective approach to enhancing diversity in the STEM workforce. This may potentially help foster a diverse talent pool of future chemistry educators, which may ultimately break the cycle of underrepresentation of minorities.

### Introductory Chemistry Instructors’ Professional Development Experiences Vary by Institution Type and Tenure Status

At the aggregate level, over half of the introductory chemistry instructors start their teaching positions with no pedagogical training. Once they are in their position, instructors engage in an average of three different types of teaching related PD with a strong preference for short-term PD, such as half-day or full-day workshops. This lack of training is astonishing considering that teaching contributes to over 50% of the academic position of these instructors (from 54.0% at Ph.D. institutions to 82.9% at A.A. institutions; see Table S20) and thus represents the primary function of these instructors at these institutions. The urgent call for graduate programs and institutions to provide pedagogical training to graduate students and instructors is not new.^36,37^ Unfortunately, it has not been heard over the strong traditional academic culture for the preparation of chemistry academics.

Disaggregation by institution type provides some nuances to this picture, however. Our findings reveal that A.A. instructors took significantly more teaching-focused coursework prior to their teaching appointment compared to Ph.D. instructors. Within institution type, we found that non-tenure-track instructors at Ph.D. institutions engaged in more PD training prior to their appointment compared to tenured instructors. Once they started their position, A.A. instructors attended significantly more types of PD compared to Ph.D. instructors. Similarly, non-tenure-track instructors at Ph.D. institutions engaged in more PD once in their appointment compared to tenured instructors, and the opposite was observed at A.A. institutions.

These results seem to indicate that the level of PD instructors engage in prior to and once in their academic appointment relates to the extent to which they can devote their efforts to teaching. For example, non-tenure-track instructors at A.A. institutions are generally made up of a high proportion of part-time contingent faculty who do not have the security of employment and receive insufficient compensation; thus, they are more likely to supplement their teaching income to cover their living expenses.^38^ This situation makes it difficult for them to find and devote time to further training.^38−40^ Tenured and tenure-track instructors at Ph.D. institutions spend more of their time on their research endeavors, which are typically better rewarded than teaching efforts.^25,36^ These assumptions should be further tested through empirical studies (e.g., national surveys and in-depth interviews) with instructors across various academic appointments and at varying institution types. Given that teaching is the core mission of any postsecondary institution, it is paramount that institutions develop policies and support mechanisms related to pedagogical training to ensure that students in introductory chemistry courses are taught by instructors who are pedagogically trained.

### Introductory Chemistry Instructors Lecture Less at A.A. Institutions but Also Use Less RBIS than Those at Ph.D. Institutions

Results from this study are in alignment with previous findings from large-scale survey studies and observational studies of STEM courses.^13−15,41^ Lecturing is the most prevalent type of instructional practice that students experience in introductory chemistry courses. Only about half of the participating instructors implement RBIS in their classes, even though most (80%) indicate knowing about these strategies. This misalignment between knowledge and usage of RBIS has been reported previously in studies on physics instructors, for example,^14^ and has led to an extensive research endeavor aimed at identifying barriers to adoption. Typical barriers reported in the literature include lack of incentives, formal training in pedagogy, and departmental norms with respect to teaching.^25,42^ This work has contributed to shifting the focus from the dissemination of active learning strategies aimed at individual instructors to changing the departmental culture around teaching and addressing the systems used to evaluate instructors.^43^ The new insight that this study provides is with respect to differences found between institution types.

In this study, A.A. instructors reported lecturing significantly less time in class compared to Ph.D. instructors, but they also reported less awareness and use of RBIS compared to Ph.D. instructors. A.A. instructors reported facilitating more whole-class discussions and having students work individually more compared to Ph.D. instructors. In prior research studies, decreased lecturing time is often thought of as an indication of a higher degree of use of active learning strategies, including RBIS. Our study suggests that it is unclear whether this assumption is accurate. A.A. instructors may be supporting their students in the construction of knowledge (one aspect of active learning) through whole-class discussion and the design of individual work, for example. Similarly, the use of many of the RBIS explored in this study should coincide theoretically with a reduction in lecture time. The fact that Ph.D. instructors report using RBIS more but also lecturing more may indicate that they preferentially use RBIS that take a small portion of class time (e.g., think-pair-share) or do not use RBIS often. The research community thus needs to identify better survey markers of active learning practices that can provide a more nuanced and accurate picture of the instruction provided and identify an active learning implementation threshold linked to positive student learning outcomes. The difference between A.A. and Ph.D. instructors is also perplexing in light of the results of pedagogical training. A.A. instructors have received more training than Ph.D. instructors yet know less about RBIS. Further research is thus warranted for understanding the nature of PD that A.A. instructors engage in.

### Non-Tenure-Track Instructors in Ph.D. Institutions Are More Knowledgeable and Higher Users of RBIS than Tenured Instructors

Comparative analyses by tenure status allowed us to see the pattern between tenured and non-tenure-track instructors at Ph.D. institutions. Our results suggest that non-tenure-track chemistry instructors are more engaged in PD and have a higher level of adoption of RBIS. Compared to their tenured counterparts, non-tenure-track respondents had more experiences with PD both prior to and after starting their teaching appointment and spent less time lecturing during class; furthermore, a larger proportion of them knew of and used RBIS. These findings diverge from prior empirical evidence collected from 1799 STEM instructors at a public research-intensive university, which suggested that tenured instructors adopt significantly more RBIS than their non-tenure-track counterparts.^28^ Moreover, two other national survey studies of physics, chemistry, and mathematics introductory instructors had found no difference by tenure status on both the proportion of class time spent lecturing and RBIS use.^17,18^ Our findings, which focused specifically on chemistry instructors, thus highlight the need to disaggregate the data by STEM discipline when exploring instructional practices. Each STEM discipline may have different norms around teaching that are attenuated and undetectable when merged with other disciplines. For example, there is often a norm in physics departments at Ph.D. institutions that anyone can teach the first-year courses, and thus, tenure-track instructors are often part of the rotating instructional team for introductory physics. This culture is absent in chemistry departments at Ph.D. institutions, where specialization in a subdiscipline is perceived as bounding to the course(s) that one can teach. This difference in culture can lead to a plethora of different instructional norms. While large-scale studies across STEM disciplines can provide institutional change agents with tools that can be broadly applicable, it is equally important to investigate the context of each STEM discipline within their institution in order to fine-tune these tools to meet the need of the local context.

Researchers in higher education noted that non-tenure-track instructors, compared to their tenure-track and tenured counterparts, generally have greater teaching loads, less access to PD resources, less engagement in decision-making in the department, and weaker job security, which may hinder their participation in PD and engagement in teaching innovations.^44,45^ Findings in our study do not fully align with these observations. Thus, future research exploring factors that motivate RBIS adoption for this group of instructors in the chemistry context could inform the development of strategies to engage non-tenure-track and tenure-track/tenured instructors in pedagogical growth. Additionally, departmental leaders should acknowledge these non-tenure-track instructors’ knowledge and practical experiences with RBIS and leverage their knowledge in promoting instructional change in their department. Lack of communication and engagement across faculty rank has been cited as one of the barriers for STEM education changes.^25^ Therefore, chemistry departmental leaders should work toward communities of practice for instructors across faculty ranks and promote regular communication about their teaching.

## Limitations

One of the main limitations of this study is the self-reported nature of the measures (e.g., class time spent on class activities, RBIS knowledge and usage, decision-makers on course components, and PD experiences). Self-reported measures may lead to decreased reliability and validity of the results. For example, instructors may be aware of the purpose of the survey questions and may choose what are perceived as socially desirable responses, such as over-reporting their use of RBIS. However, in terms of teaching practices, research has demonstrated that self-reported data align well with observational data.^46,47^

Furthermore, sample sizes vary across tenure statuses, which may be an indication of the existing sampling bias in this study (i.e., faculty members teaching introductory chemistry courses). Tenure-track instructors are a smaller proportion of our sample compared to tenured instructors and non-tenure-track instructors, especially at Ph.D. institutions, which may limit the generalizability of the findings for tenure-track instructors. It should also be noted that in the current study, we did not differentiate between instructors with non-benefit-eligible part-time contingent appointments and benefit-eligible full-time contingent appointments associated with a one or multiyear contract. These instructors may have varying employment security, teaching load, and opportunities to PD, resulting in differences on reported PD experiences and adoption of active learning. Thus, future studies may provide nuanced insights by differentiating different working conditions for non-tenure-track instructors.

Finally, the study was conducted in the pre-pandemic era and only includes instructors who taught in-person classes at that time (2017–18 and 2018–19). It is possible that the pandemic and the required shift to online courses have contributed to shifts in the instructors’ classroom pedagogy. Thus, we acknowledge that the generalizability of the results may not be extended to the online teaching setting and may be limited in the post-pandemic era.

## Conclusions

This study explored the characteristics of instructors and the landscape of active learning implementation in introductory chemistry courses. Our findings show that instructors in these courses are not diverse in terms of gender and race/ethnicity, with males of European decent being prominent. Instructors enter their teaching positions with minimal pedagogical training. While they participate in more training once in their position, these trainings are often short (e.g., half-day). With respect to teaching, we found that while the majority of chemistry instructors have knowledge of RBIS, their consistent implementation remains limited, with lecturing still being the instructional practice of choice. Interestingly, we found differences between institution types and across tenure statuses within institutions in terms of pedagogical training and the use of active learning. These findings underscore the importance of fostering communication about teaching practices across institutions and tenure statuses. Our findings provide a baseline for future studies that aim to assess the effectiveness of interventions fostering the implementation of active learning in introductory chemistry courses.

## Supporting Information Available

Survey questions and additional demographic data and survey results (PDF, DOCX)
