# Supplementary material for: A National Snapshot of Introductory Chemistry Instructors and Their Instructional Practices
Source: J Chem Educ. 2024 Mar 13;101(4):1457–68. doi: 10.1021/acs.jchemed.4c00040 (PMC11008091; doi:10.1021/acs.jchemed.4c00040)
Supplement: Supplementary file 1 — ed4c00040_si_001.pdf [file ed4c00040_si_001.pdf]

Supplemental materials for

A National Snapshot of Introductory Chemistry Instructors and Their Instructional Practices

Ying Wang,[1] Naneh Apkarian,[2] Melissa H. Dancy,[3] Charles Henderson,[4] Estrella Johnson,[5] Jeffrey R. Raker,[6,7] Marilyne Stains[1]*

Affiliations

1. Department of Chemistry, University of Virginia, Charlottesville, VA 22904-4319, USA.
2. School of Mathematical and Statistical Sciences, Arizona State University, Tempe, AZ, 85287-1804, USA.
3. The Evaluation Center, Western Michigan University, Kalamazoo, MI 49008-5252, USA.
4. Department of Physics and Mallinson Institute for Science Education, Western Michigan University, Kalamazoo, MI 49008-5252, USA.
5. Department of Mathematics, Virginia Polytechnic Institute and State University, Blacksburg, VA 24061-0123, USA.
6. Department of Chemistry, University of South Florida, Tampa, FL 33620-5250, USA.
7. Center for the Improvement of Teaching and Research on Undergraduate STEM Education, University of South Florida, Tampa, FL 33620-5250, USA.

*Corresponding author. E-mail: mstains@virginia.edu

Survey

Course Enrollment

(8) What is the approximate enrollment in a typical lecture section of your course?

Time allocation in four class activities

(14) During a typical week, what proportion of time during regular class meetings (i.e., lecture sections) do students spend doing the following?

Working individually	0% ▾
Working in small groups	0% ▾
Participating in whole-class discussions	0% ▾
Listening to the instructor lecture or solve problems	0% ▾

General RBIS usage and knowledge

(18) Please indicate no or yes to the following statements about your awareness/usage of RBIS:

	No	Yes
Prior to this survey, I already knew about RBIS.	○	○
I have thought about how to implement RBIS in my courses.	○	○
I've spent time learning about RBIS (e.g.,	○	○

attended workshop, experimented in class, read education literature) and I am prepared to use them.

I consistently use RBIS in my courses.	◯	◯
I consistently use RBIS and I continue to learn about and experiment with new RBIS	◯	◯
I have evidence that my teaching has improved since I started using RBIS.	◯	◯

Specific RBIS usage and knowledge

An alphabetical list of selected RBIS (research-based instructional strategies) is available here. We are curious which of these you have heard of and which, if any, you have tried using. Please indicate your awareness and (if applicable) usage of each of these instructional strategies, which will be presented one at a time.

(21.a) **Chemical Thinking: A curriculum which is designed to introduce college students to chemistry as a powerful way of thinking with multiple applications in critical areas.**

- ◯ I have never heard of this
- ◯ I know the name, but not much more
- ◯ I know about this, but have never used it in this course
- ◯ I have tried it in this course, but no longer use it
- ◯ I currently use it in this course to some extent

(21.b) **<u>Chemistry, Life, the Universe, and Everything (CLUE):</u> A transformed general chemistry curriculum that focuses on scaffolded progressions around four core ideas: structure and properties, bonding and interactions, energy, and change and stability.**

- I have never heard of this
- I know the name, but not much more
- I know about this, but have never used it in this course
- I have tried it in this course, but no longer use it
- I currently use it in this course to some extent

(21.c) **<u>Concept Inventories:</u> Multiple-choice assessments which cannot be answered via calculation, only through conceptual understanding (e.g., Force Concept Inventory, Calculus Concept Inventory, Chemistry Concept Inventory).**

- I have never heard of this
- I know the name, but not much more
- I know about this, but have never used it in this course
- I have tried it in this course, but no longer use it
- I currently use it in this course to some extent

(21.d) **<u>Concept Maps:</u> Students generate diagrams that describe processes, concepts, and the interrelationships between them for a particular content area.**

- I have never heard of this
- I know the name, but not much more
- I know about this, but have never used it in this course
- I have tried it in this course, but no longer use it
- I currently use it in this course to some extent

(21.e) **<u>Flipped Classroom:</u> Primary mode of delivery of content occurs outside of the classroom (i.e., using videos, textbook, activities) and the application of content occurs inside the classroom.**

- I have never heard of this
- I know the name, but not much more
- I know about this, but have never used it in this course
- I have tried it in this course, but no longer use it
- I currently use it in this course to some extent

(21.f) **<u>Formal Small Group Work:</u> Use of assigned small groups in which individuals are responsible for their own learning as well as that of others in the group (e.g., collaborative/cooperative learning).**

- I have never heard of this
- I know the name, but not much more
- I know about this, but have never used it in this course
- I have tried it in this course, but no longer use it
- I currently use it in this course to some extent

(21.g) **<u>Inquiry-Based Learning (IBL):</u> A broad range of empirically validated teaching methods which emphasize (a) deeply engaging students and (b) providing students with opportunities to authentically learn by collaborating with their peers.**

- I have never heard of this
- I know the name, but not much more
- I know about this, but have never used it in this course
- I have tried it in this course, but no longer use it
- I currently use it in this course to some extent

(21.h) **<u>Interactive Lecture Demonstrations:</u> A pre-lecture activity wherein students *predict* the outcome of a demonstration, *experience* the demonstration, and *reflect* on the outcome and how it compares to their initial prediction**

- I have never heard of this
- I know the name, but not much more
- I know about this, but have never used it in this course
- I have tried it in this course, but no longer use it
- I currently use it in this course to some extent

(21.i) **<u>Just-in-Time Teaching:</u> Students complete a pre-class assignment which the instructor reviews before class, adjusting class emphasis and discussion based on students' responses.**

- I have never heard of this
- I know the name, but not much more
- I know about this, but have never used it in this course
- I have tried it in this course, but no longer use it
- I currently use it in this course to some extent

(21.j) **<u>Peer Instruction:</u> The instructor poses a question, after which students are given time to reflect and select the answer. The instructor reviews these responses while students discuss their thinking and commit to a (possibly new) answer - the instructor then reviews these to decide whether or not students are ready to move on.**

- I have never heard of this
- I know the name, but not much more
- I know about this, but have never used it in this course
- I have tried it in this course, but no longer use it
- I currently use it in this course to some extent

(21.k) **<u>Peer-Led Team Learning (PLTL):</u> Students who have previously taken (and succeeded) in the course act as peer-leaders, leading weekly problem-solving sessions related to course material.**

- I have never heard of this
- I know the name, but not much more
- I know about this, but have never used it in this course
- I have tried it in this course, but no longer use it
- I currently use it in this course to some extent

(21.l) **Peer-Reviewed Scientific Writing: Students submit written assignments including term papers and laboratory reports that are peer-reviewed before submitting final versions (e.g., Calibrated Peer Review, MyReviewers).**

- I have never heard of this
- I know the name, but not much more
- I know about this, but have never used it in this course
- I have tried it in this course, but no longer use it
- I currently use it in this course to some extent

(21.m) **Process Oriented Guided Inquiry Learning (POGIL):**

Students work collaboratively in small groups (3-4) on tasks in class, with a facilitator present. The activities are specifically designed for POGIL and are the first introduction to the content in class.

- I have never heard of this
- I know the name, but not much more
- I know about this, but have never used it in this course
- I have tried it in this course, but no longer use it
- I currently use it in this course to some extent

(21.n) **Ranking Task Exercises in Physics and/or TIPERs: Activities asking students to make comparative judgements about variations on a particular physics situation; Tasks Inspired by Physics Education Research.**

- I have never heard of this
- I know the name, but not much more
- I know about this, but have never used it in this course
- I have tried it in this course, but no longer use it
- I currently use it in this course to some extent

(21.o) **<u>Reform-Oriented Textbooks:</u> Textbooks aligned with the Calculus Reform movement which emphasize modeling, multiple representations, and/or exploration (e.g., Hughes-Hallett et al., Harvard Calculus).**

- I have never heard of this
- I know the name, but not much more
- I know about this, but have never used it in this course
- I have tried it in this course, but no longer use it
- I currently use it in this course to some extent

(21.p) **<u>Studio/SCALE-UP:</u> Students work in small groups on hands-on activities, simulations, interesting questions or problems for the majority of the class period.**

- I have never heard of this
- I know the name, but not much more
- I know about this, but have never used it in this course
- I have tried it in this course, but no longer use it
- I currently use it in this course to some extent

(21.q) **<u>Teaching with Computer Simulations and Interactive Animations:</u> Interactive computer animations, in which variables of the system or other aspects can be manipulated, are used to supplement classroom instruction (e.g., Phet simulations, Desmos).**

- I have never heard of this
- I know the name, but not much more
- I know about this, but have never used it in this course
- I have tried it in this course, but no longer use it
- I currently use it in this course to some extent

(21.r) Think-Pair-Share: Posing a problem or question, having students work on it individually for a short time and then forming pairs and reconciling their solutions, followed by whole classroom discussion of students' responses.

- I have never heard of this
- I know the name, but not much more
- I know about this, but have never used it in this course
- I have tried it in this course, but no longer use it
- I currently use it in this course to some extent

(21.s) Tutorials in Introductory Physics: Lecture tool where students complete a written activity during or after lecture, often in small groups.

- I have never heard of this
- I know the name, but not much more
- I know about this, but have never used it in this course
- I have tried it in this course, but no longer use it
- I currently use it in this course to some extent

Professional development experiences

(33) How many academic courses focused on learning how to teach have you taken at the undergraduate, graduate, and postdoctoral levels?

select ▼

(34) **Have you ever participated in any of the following types of teaching-related professional development?**

	No	Yes
Half-day workshop(s)	○	○
Full-day or longer workshop(s)	○	○
Attending a teaching-focused conference	○	○
Regular meetings as part of a formal program (e.g., learning community)	○	○
New faculty experience at my institution	○	○
New faculty workshop external to my institution (e.g., Cottrell Scholars Collaborative - CSC NFW, Project NExT, Project ACCESS, Physics New Faculty Workshop)	○	○
Other: ________	○	○

Academic Rank

(38) What is your present academic rank?

- ○ Professor
- ○ Associate Professor
- ○ Assistant Professor
- ○ Lecturer or Instructor
- ○ Visiting Professor, Lecturer, or Instructor
- ○ Postdoctoral Instructor
- ○ Graduate Student Instructor or Teaching Assistant

Tenure Status

(39) What is your tenure status at this institution?

- ○ Tenured
- ○ On tenure track, but not tenured
- ○ Not on tenure track, but this institution has a tenure system
- ○ No tenure system at this institution

Distribution of position

(43) **What is the approximate distribution of your position at $institution?**

**Research**	0% ▼
**Teaching**	0% ▼
**Service**	0% ▼
**Administration**	0% ▼
**Other**	0% ▼

Teaching Load

(45) **What is your typical teaching load (i.e., how many course sections do you teach) during a single term?**

select ▼ Course sections

Teaching Experiences

(48) **For how many years have you taught $discipline3 courses...**

At the postsecondary (college) level? select ▼

At the secondary (high school) level? select ▼

Demographics

(55) How would you describe yourself? (Select all that apply)

Note: We recognize that the options presented below are imperfect and gloss over much of the nuance associated with gender identity and gender expression. In order to be able to compare our results with other large data sets, we offer the terms used most frequently in survey work. We recognize the limitations of such a list.

- ☐ Woman
- ☐ Man
- ☐ Transgender
- ☐ Cisgender
- ☐ Gender fluid
- ☐ Agender
- ☐ Not Listed (please specify): ______
- ☐ Prefer not to answer

(56) How would you describe yourself? (Select all that apply)

Note: We recognize that the options presented below are imperfect and gloss over much of the nuance associated with race and ethnic identity. In order to be able to compare our results with other large data sets, we retain the major categories used on the US census. We have included additional options, noted within the headings used by the US census, but recognize the limitations of such a list.

- ☐ American Indian and/or Alaska Native
- Asian
  - ☐ Central Asian
  - ☐ East Asian
  - ☐ South Asian
  - ☐ Southeast Asian
- ☐ Black and/or African American
- ☐ Hispanic and/or Latinx
- ☐ Native Hawaiian and/or other Pacific Islander
- White
  - ☐ European
  - ☐ Middle Eastern and/or North African
- ☐ Not listed (please specify): ______
- ☐ Prefer not to answer

Table S1: Gender by Tenure Status by Institution Type

Institution Type	Percentage of Total Participants within each Tenure Status in each Institution Type (%)							
	Tenured		On tenure track		Not on a tenure track		No tenure system	
	Women	Men	Women	Men	Women	Men	Women	Men
A.A.	46.3	53.7	47.6	52.4	38.1	61.9	53.6	46.4
B.A./B.S.	35.3	64.7	45.3	54.7	75.0	25.0		
M.A./M.S.	41.9	58.1	40.5	59.5	45.5	54.5		
Ph.D.	18.5	81.5	40.0	60.0	53.4	46.6		

Table S2: Race/Ethnicity by Tenure Status by Institution Type

Institution Type	Percentage of Total Participants within each Tenure Status in each Institution Type (%)							
	Tenured		On tenure track		Not on a tenure track		No tenure system	
	European	Non-European	European	Non-European	European	Non-European	European	Non-European
A.A.	77.9	22.1	63.6	36.4	72.1	27.9	91.4	8.6
B.A./B.S.	91.2	8.8	84.1	15.9	76.2	23.8		
M.A./M.S.	79.3	20.7	56.4	43.6	76.7	23.3		
Ph.D.	79.2	20.8	88.0	12.0	81.9	18.1		

Table S3: Instructors' Highest Degree by Institution Type

Degree level	Percentage of Total Participants in each Institution Type (%)			
	A.A. (n=343)	B.A./B.S. (n=342)	M.A./M.S. (n=151)	Ph.D. (n=247)
Bachelor's level degree	1.5	0.0	0.0	0.8
Master's level degree	32.4	2.6	7.9	5.7
Doctoral level degree	66.2	97.4	92.1	93.5

Table S4: Teaching Experiences at Postsecondary Level by Institution Type

Years of Teaching	Percentage of Total Participants in each Institution Type (%)			
	A.A. (n=342)	B.A./B.S. (n=340)	M.A./M.S. (n=149)	Ph.D. (n=245)
1-4	10.5	15.6	19.5	14.7
5-9	19.6	15.3	26.8	17.1
10-14	21.6	18.2	14.8	16.7
15 and 15+	48.2	50.9	38.9	51.4

Table S5. Number of Courses Taught Each Semester by Institution Type

Institution Type	n	Mean	SE
A.A.	337	3.1	0.1
B.A./B.S.	340	3.2	0.0
M.A./M.S.	150	2.9	0.1
Ph.D.	246	1.9	0.1

Table S6. Enrollment in Class by Institution Type

Institution Type	n	Mean	SE
A.A.	415	30.4	0.8
B.A./B.S.	379	47.5	1.7
M.A./M.S.	167	79.1	4.1
Ph.D.	271	193.9	8.2

Table S7. Distributions of Decision Makers on Content and Topic Coverage, Textbook, Exams, and Instructional Methods

	Percentage of Total Participants in each Institution Type (%)		
	Myself	Myself and others	One or more other people
Instructional Methods	85.3	14.0	0.7
Exams	83.2	15.6	1.2
Content and Topic Coverage	19.6	68.8	11.6
Textbook	18.0	64.4	17.6

Table S8. Distributions of Decision Makers on Instructional Methods by Institution Type

	Percentage of Total Participants in each Institution Type (%)		
	Myself	Myself and others	One or more other people
A.A.	89.4	10.2	0.5
B.A./B.S.	88.3	11.7	-
M.A./M.S.	80.8	18.6	0.6
Ph.D.	77.8	20.4	1.9

Table S9. Distributions of Decision Makers on Exam by Institution Type

	Percentage of Total Participants in each Institution Type (%)		
	Myself	Myself and others	One or more other people
A.A.	92.0	7.0	1.0
B.A./B.S.	84.6	15.1	0.3
M.A./M.S.	81.3	16.3	2.4
Ph.D.	68.9	28.9	2.2

Table S10. Distributions of Decision Makers on Content by Institution Type

	Percentage of Total Participants in each Institution Type (%)		
	Myself	Myself and others	One or more other people
A.A.	27.1	55.8	17.1
B.A./B.S.	17.0	79.1	4.0
M.A./M.S.	11.5	71.1	17.5
Ph.D.	16.7	73.0	10.4

Table S11. Distributions of Decision Makers on Textbook by Institution Type

	Percentage of Total Participants in each Institution Type (%)		
	Myself	Myself and others	One or more other people
A.A.	28.9	48.4	22.7
B.A./B.S.	13.6	77.1	9.3
M.A./M.S.	10.2	69.3	20.5
Ph.D.	12.3	68.0	19.7

Table S12. Distributions of RBIS Knowers and Users by Institution

	Percentage of Total Participants within each Tenure Status (%)	
	Knowers	Knowers and Users
A.A.	73.1	43.6
B.A./B.S.	85.8	55.4
M.A./M.S.	80.3	53.5
Ph.D.	79.4	53.3

Table S13. Distributions of RBIS Knowers and Users by Tenure Status at A.A. Institutions

	Percentage of Total Participants within each Tenure Status (%)	
	Knowers	Knowers and Users
No tenure system	66.2	30.4
Not on tenure-track	45.3	29.0
Tenure-track	79.1	50.0
Tenured	80.0	46.0

Table S14. Distributions of RBIS Knowers and Users by Tenure Status at B.A./B.S. Institutions

	Percentage of Total Participants within each Tenure Status (%)	
	Knowers	Knowers and Users
Not on tenure-track	75.0	52.5
Tenure-track	84.4	59.4
Tenured	87.1	52.2

Table S15. Distributions of RBIS Knowers and Users by Tenure Status at M.A./M.S. Institutions

	Percentage of Total Participants within each Tenure Status (%)	
	Knowers	Knowers and Users
Not on tenure-track	76.1	53.5
Tenure-track	81.1	55.6
Tenured	78.3	45.8

Table S16. Distributions of RBIS Knowers and Users by Tenure Status at Ph.D. Institutions

	Percentage of Total Participants within each Tenure Status (%)	
	Knowers	Knowers and Users
Not on tenure-track	87.8	64.4
Tenure-track	76.0	68.0
Tenured	68.2	33.0

Table S17. Distribution of Instructors in terms of their Knowledge and Usage of Specific RBIS

	Percentage of Total Participants (%)				
	I have never heard of this	I know the name, but not much more	I know about this, but have never used it in my course	I have tried it in this course, but no longer use it	I currently use it in this course to some extent
Formal Small Group Work	7.8	6.0	33.5	10.0	42.7
Flipped Classroom	4.0	2.6	54.2	10.2	29.1
Think-Pair-Share	17.7	8.8	28.9	6.7	37.9
Peer-Led Team Learning (PLTL)	12.4	8.9	39.1	8.0	31.5
Teaching with Computer Simulations and Interactive Animations	14.6	11.2	30.3	6.0	37.8
Peer Instruction	14.1	12.2	34.5	7.2	32.1
Process Oriented Guided Inquiry Learning (POGIL)	12.8	8.0	49.5	10.5	19.1
Interactive Lecture Demonstrations	19.9	9.4	35.5	7.0	28.1
Concept Maps	16.5	10.6	44.8	8.1	20.0
Just-in-Time Teaching	23.5	11.6	42.0	6.6	16.4
Peer-Reviewed Scientific Writing	22.1	12.4	53.3	5.8	6.5
Concept Inventories	37.0	13.4	24.3	4.7	20.7
Studio/SCALE-UP	47.8	9.5	27.1	2.1	13.5
Chemical Thinking	60.4	13.7	8.7	1.2	15.9
Chemistry, Life, the Universe, and Everything (CLUE)	57.5	13.3	18.8	0.6	9.8

Table S18: Descriptive Statistics of Percentage of Time Spent on Four Class Activities by Institution Type

Institution Type	Percent of Time (%)			
	Participating in whole class discussions	Working individually	Working in small groups	Listening to the instructor lecture or solve problems
A.A.	13.9 ± 3.4	13.3± 3.2	17.9± 4.2	55.0± 5.1
B.A./B.S.	9.8± 3.2	11.1± 2.8	21.5± 4.7	57.6± 5.5
M.A./M.S.	9.5± 3.8	11.2± 3.4	18.6± 4.9	60.7± 5.7
Ph.D.	8.8± 3.3	10.3± 3.1	14.8± 4.3	66.1± 5.2

Table S19: Statistics of Comparison Analysis on Percentage of Time Spent on Four Class Activities by Tenure Status by Institution

Institution Type	Tenured	On tenure track	Not on tenure track	No tenure system	F value	p-value	η^2
A.A.	53.9 ± 2.0	53.3 ± 3.5	57.2 ± 2.0	58.5 ± 2.8	0.832	0.477	0.008
B.A./B.S.	58.4 ± 1.8	58.6 ± 2.6	53.1 ± 4.5	-	0.761	0.468	0.005
M.A./M.S.	63.9 ± 3.1	56.4 ± 4.0	61.1 ± 4.0	-	0.999	0.371	0.014
Ph.D.	69.5 ± 2.3	73.0 ± 3.4	58.4 ± 2.4	-	7.420	0.001	0.062

Table S20: Distribution of Academic Position by Institution Type

	AA	BA/BS	MA/MS	PhD
Research	2.0 ± 0.3	18.4 ± 0.7	20.8 ± 1.4	25.8 ± 1.5
Teaching	82.9 ± 1.0	63.4 ± 0.9	60.5 ± 2.0	54.0 ± 1.7
Service	8.4 ± 0.5	13.3 ± 0.4	12.2 ± 0.7	12.4 ± 0.6

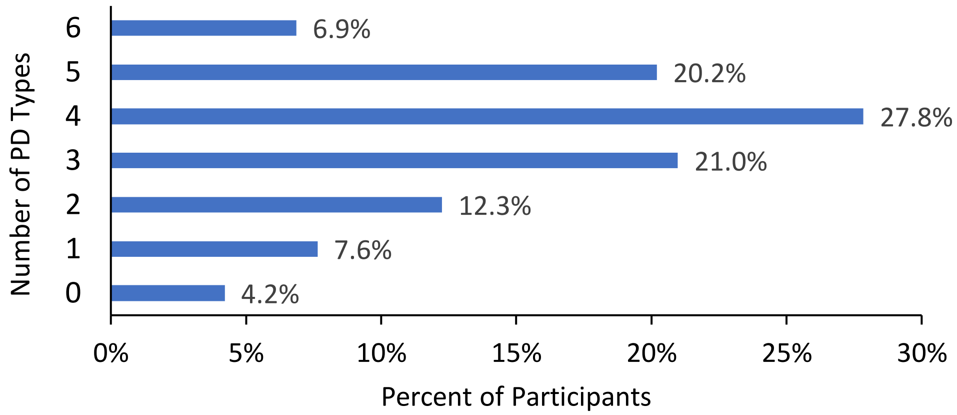

Figure S1: Distribution of Instructors by Number of Professional Development Types Attend in their Teaching Positions

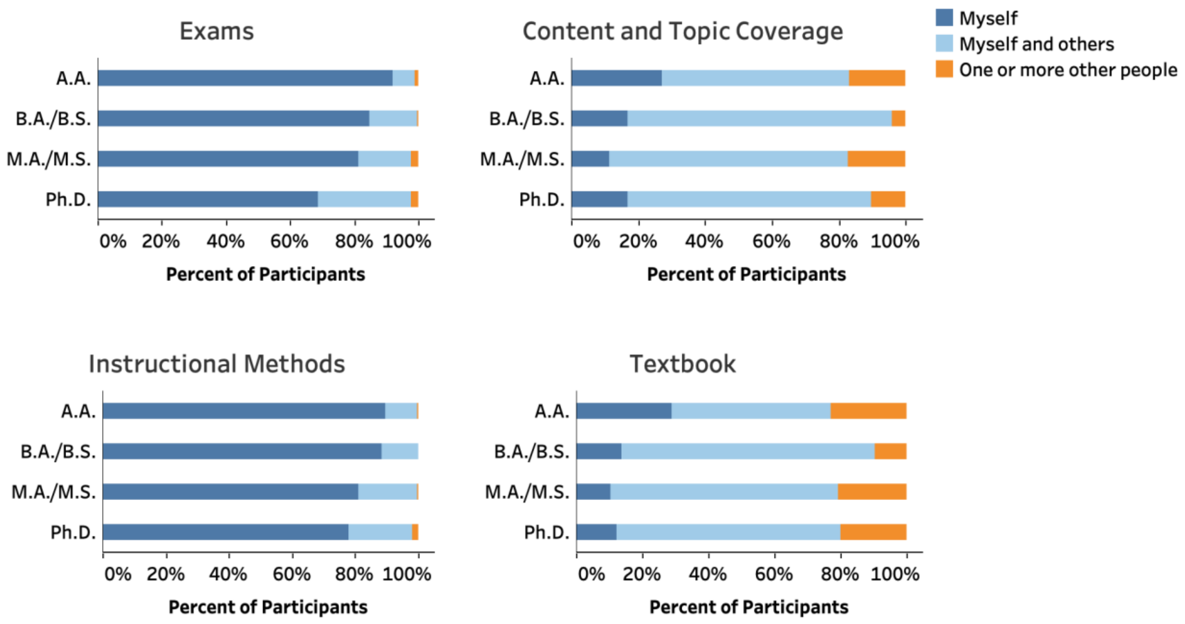

Figure S2: Distributions of Decision Makers for Four Course Components by Institution Type (A.A.: n=414, B.A./B.S.: n=377, M.A./M.S.: n =167, Ph.D.: n=270)

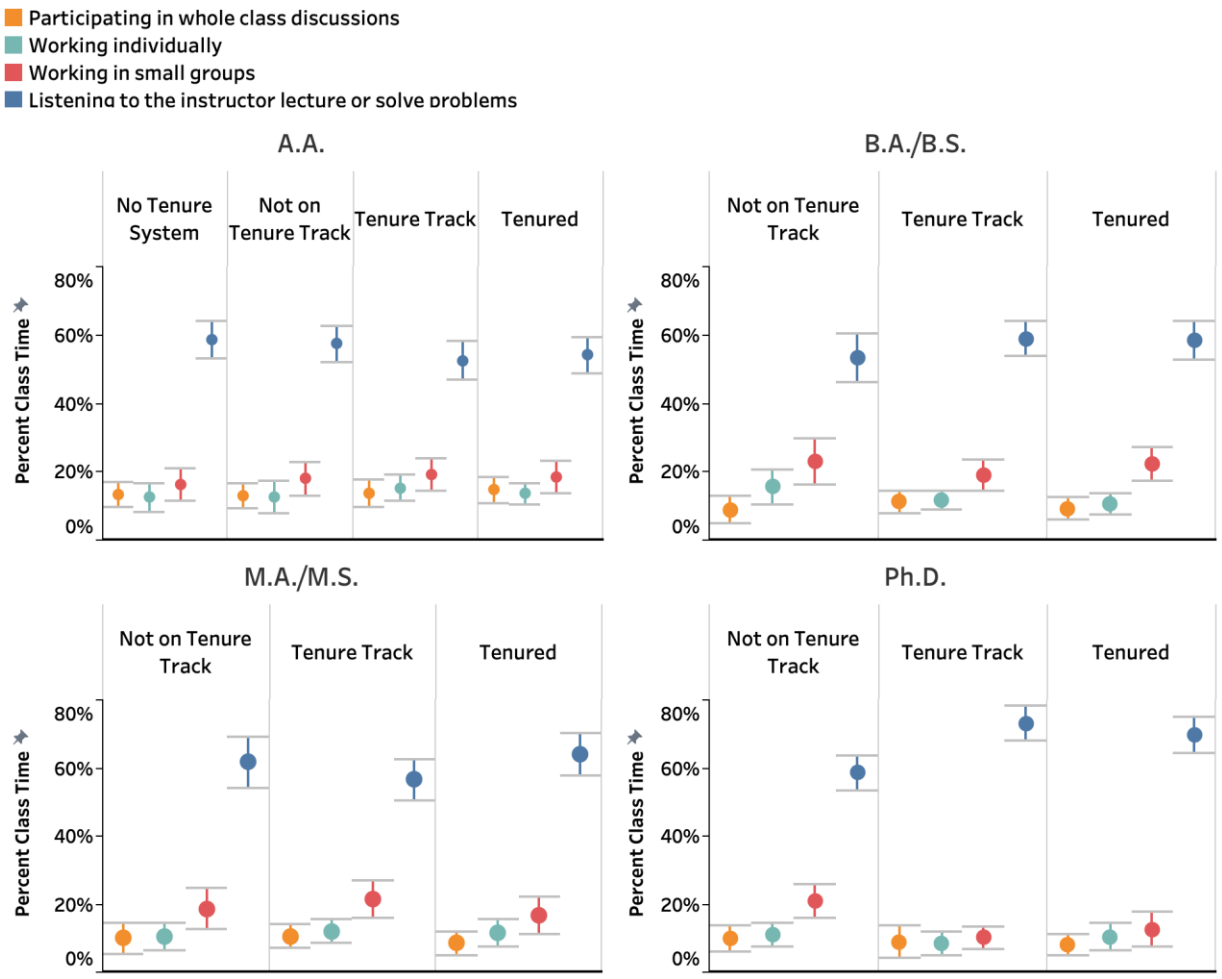

Figure S3: Percentage of Time Spent on Four Class Activities by Tenure Status by Institution
