# Supplementary material for: A National Snapshot of Introductory Chemistry Instructors and Their Instructional Practices
Source: J Chem Educ. 2024 Mar 13;101(4):1457–68. doi: 10.1021/acs.jchemed.4c00040 (PMC11008091; doi:10.1021/acs.jchemed.4c00040)
Supplement: Supplementary file 2 — ed4c00040_si_002.docx [file ed4c00040_si_002.docx]

Supplemental materials for

A National Snapshot of Introductory Chemistry Instructors and Their Instructional Practices

Ying Wang,^1^ Naneh Apkarian,^2^ Melissa H. Dancy,^3^ Charles Henderson,^4^ Estrella Johnson,^5^ Jeffrey R. Raker,^6,7^ Marilyne Stains^1^*

Affiliations

1. Department of Chemistry, University of Virginia, Charlottesville, VA 22904-4319, USA.
2. School of Mathematical and Statistical Sciences, Arizona State University, Tempe, AZ, 85287-1804, USA.
3. The Evaluation Center, Western Michigan University, Kalamazoo, MI 49008-5252, USA.
4. Department of Physics and Mallinson Institute for Science Education, Western Michigan University, Kalamazoo, MI 49008-5252, USA.
5. Department of Mathematics, Virginia Polytechnic Institute and State University, Blacksburg, VA 24061-0123, USA.
6. Department of Chemistry, University of South Florida, Tampa, FL 33620-5250, USA.
7. Center for the Improvement of Teaching and Research on Undergraduate STEM Education, University of South Florida, Tampa, FL 33620-5250, USA.

*Corresponding author. E-mail: mstains@virginia.edu

Survey

**Course Enrollment**


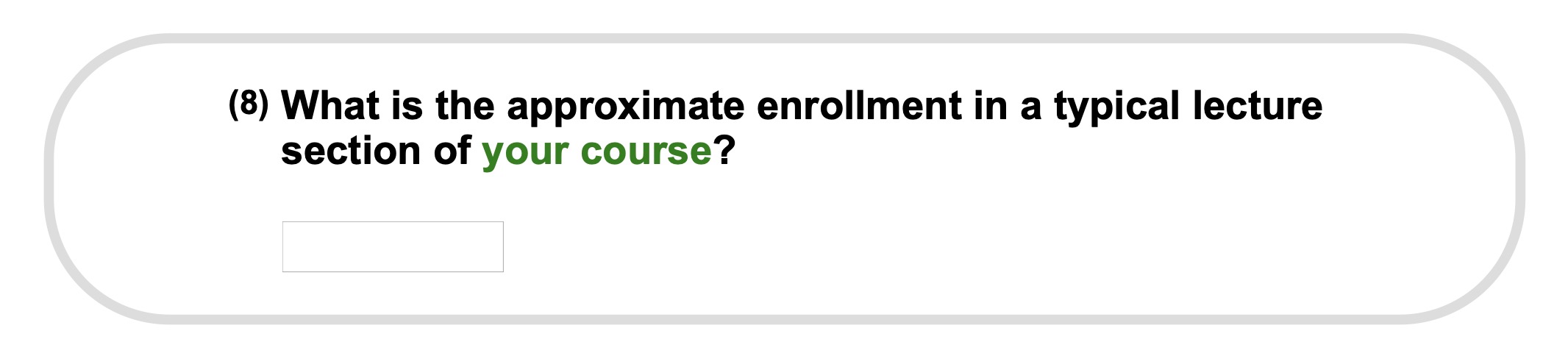


**Time allocation in four class activities**


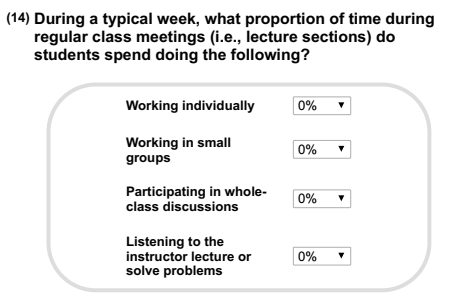


**General RBIS usage and knowledge**


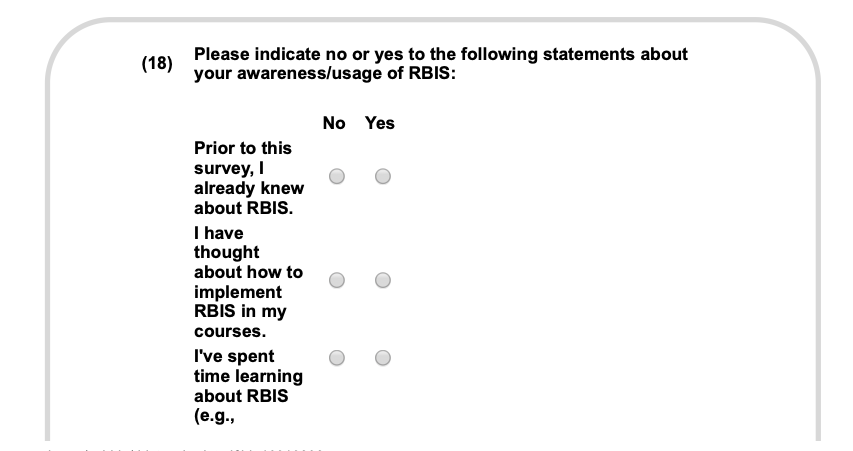


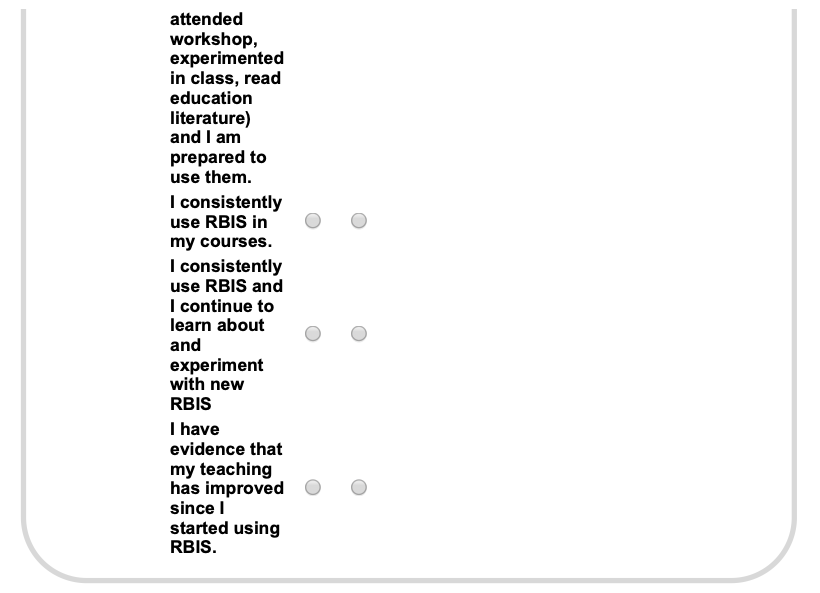


**Specific RBIS usage and knowledge**


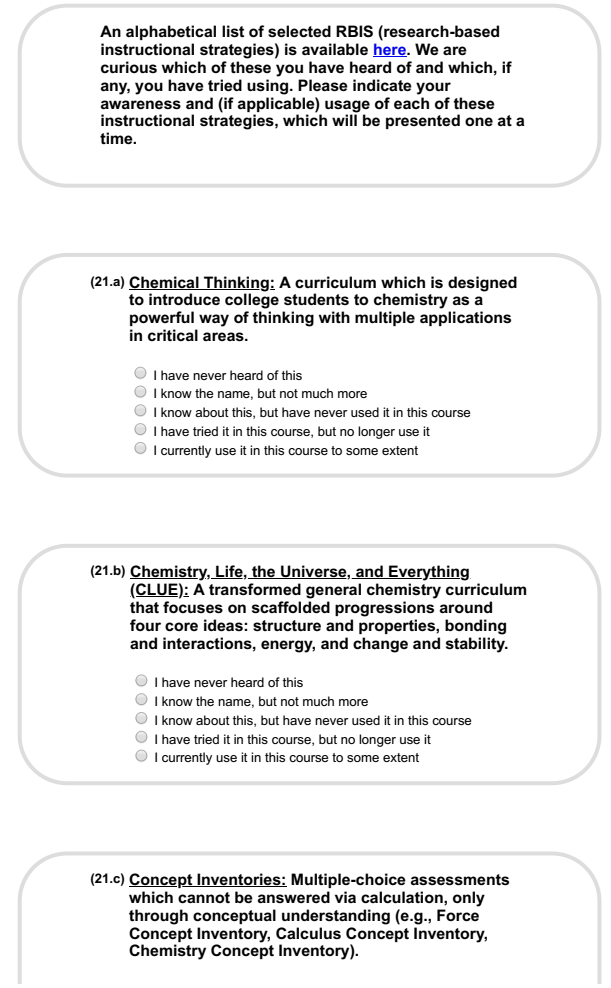


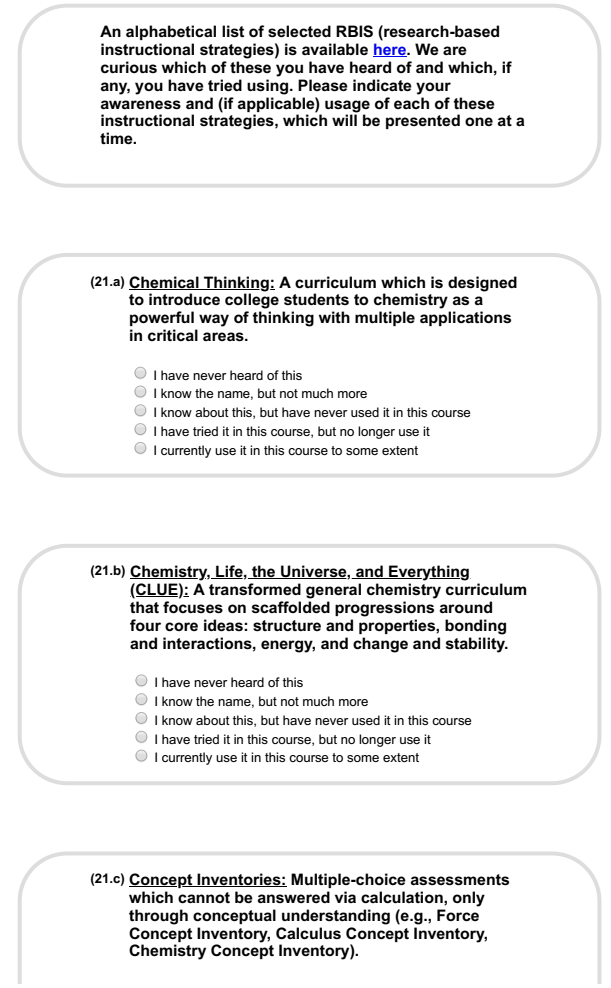


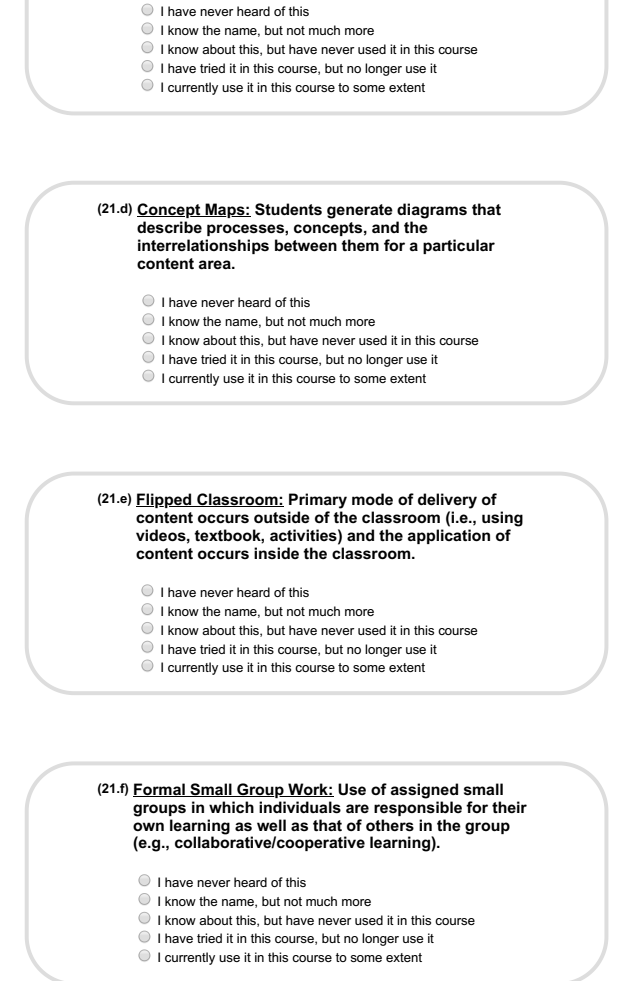


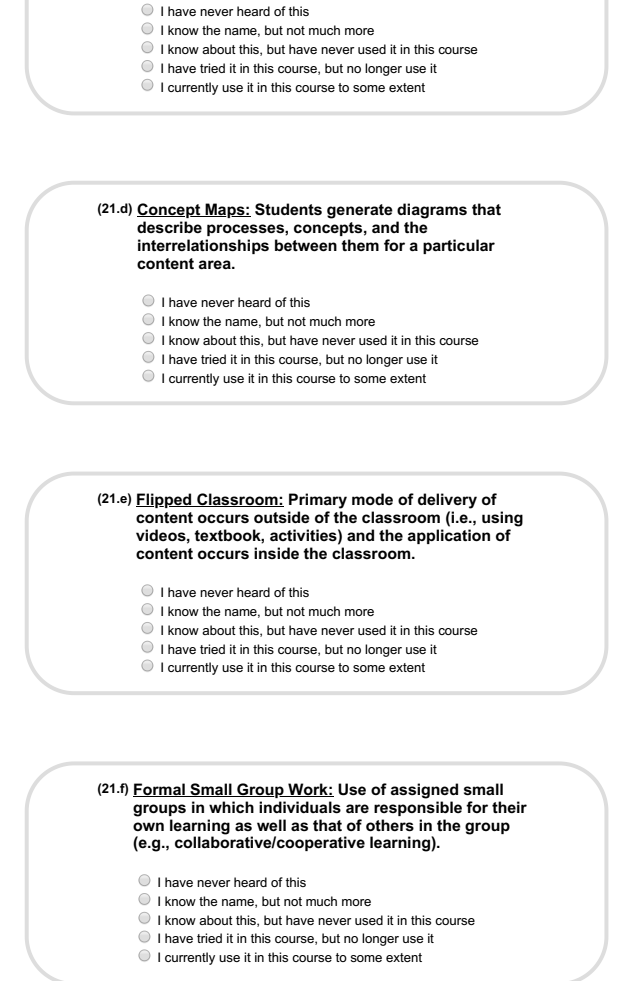

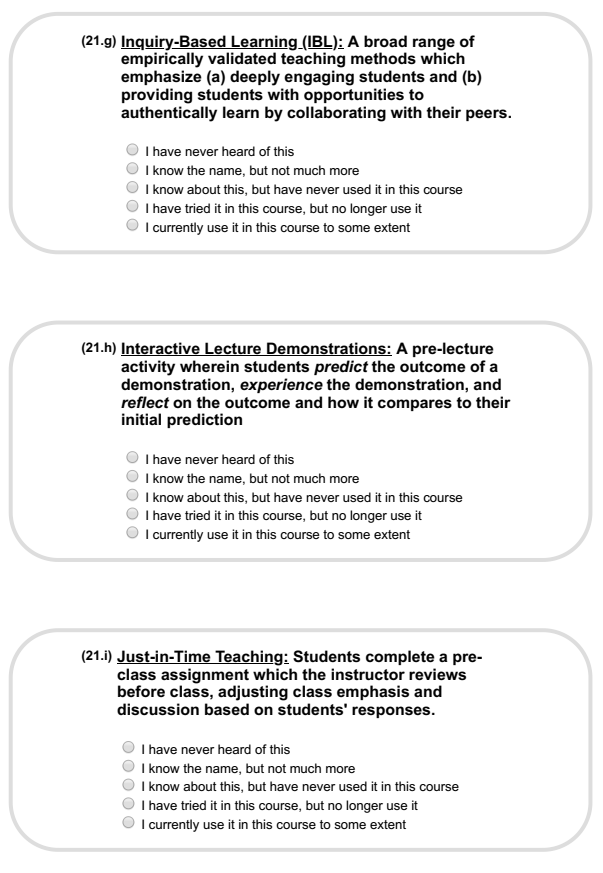

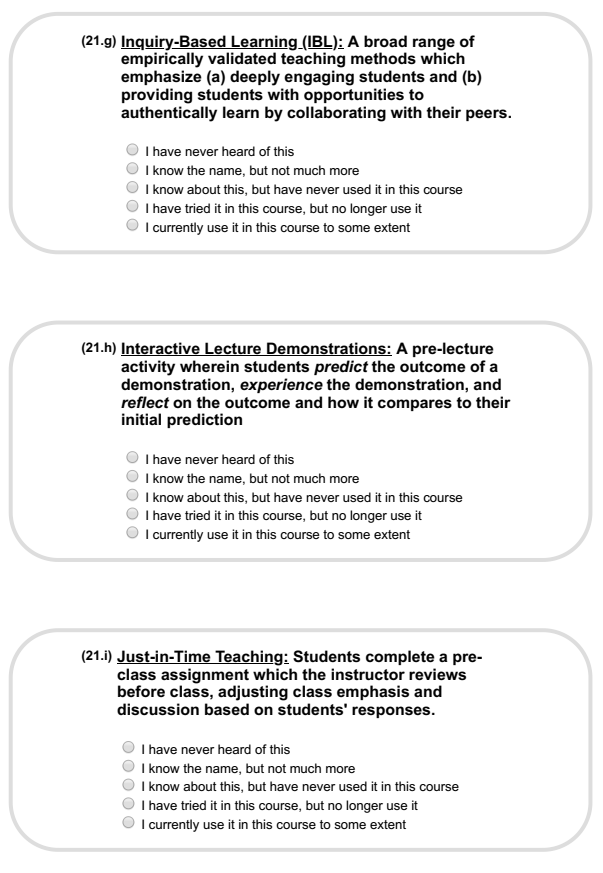


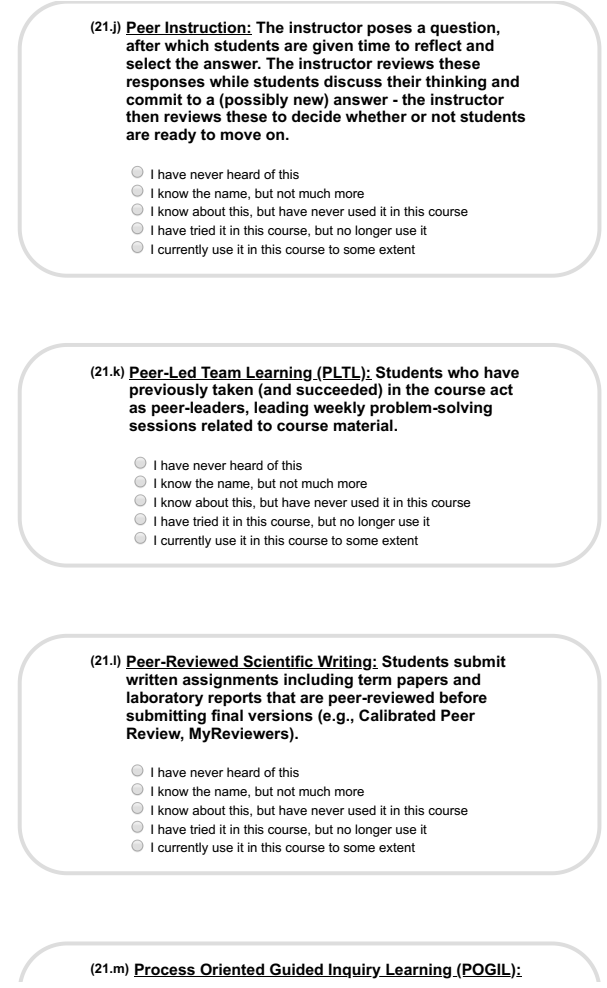


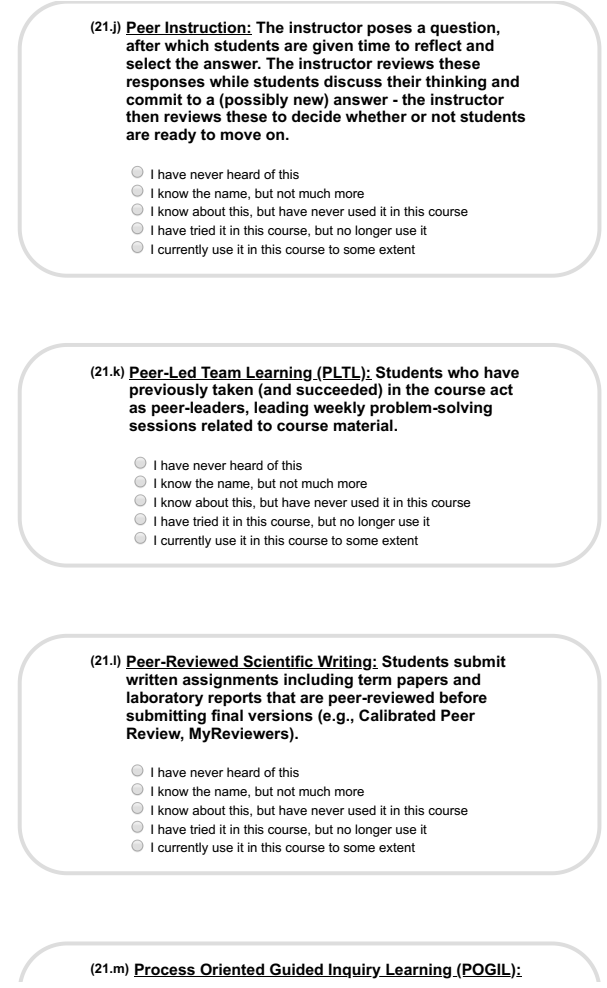


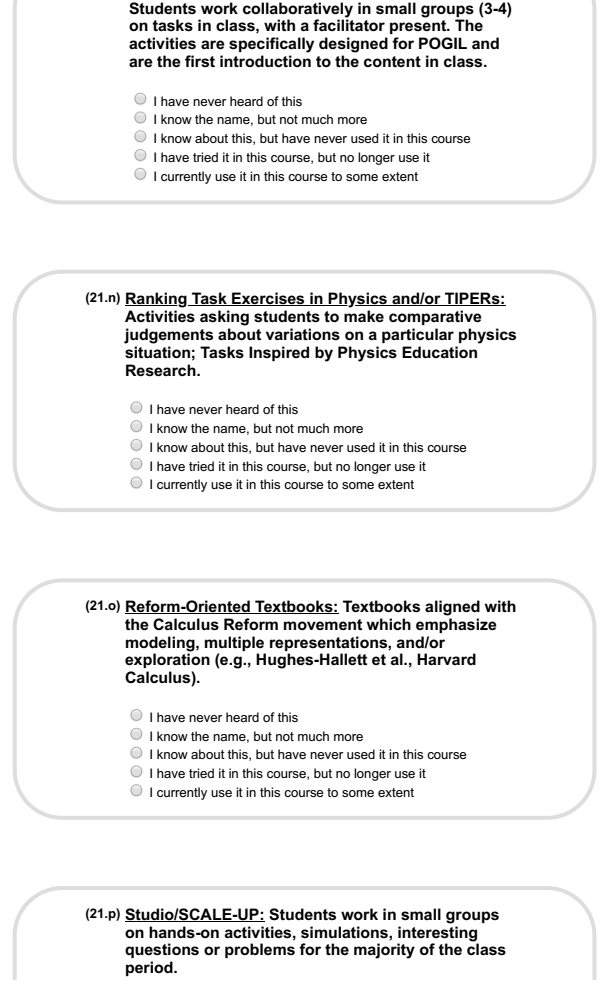


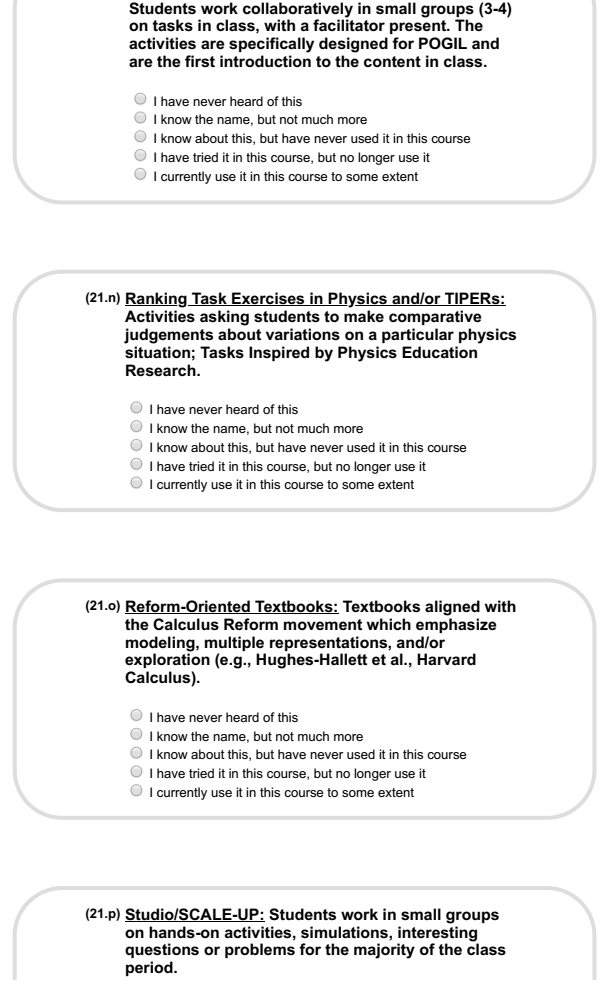


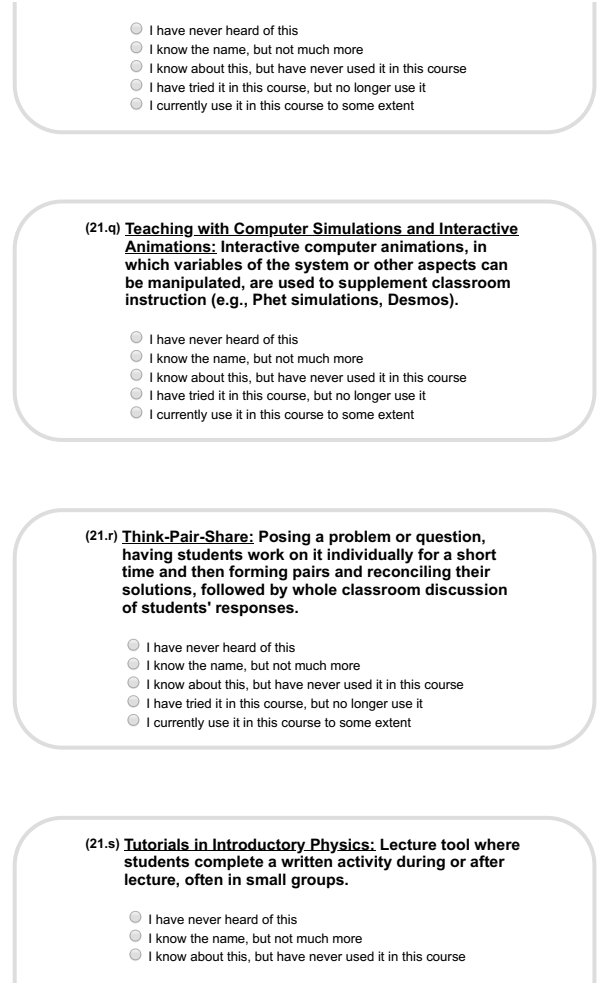


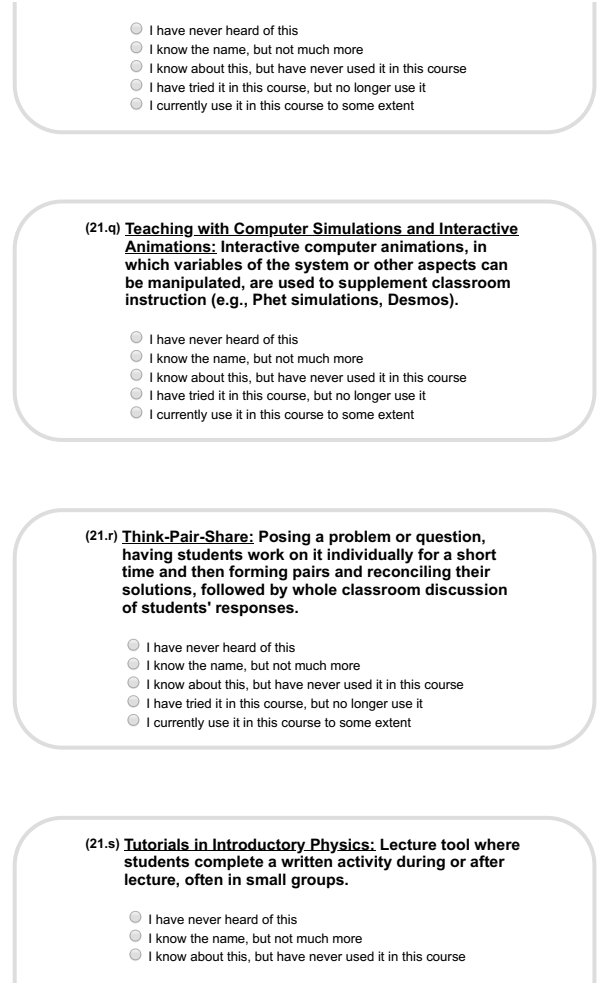


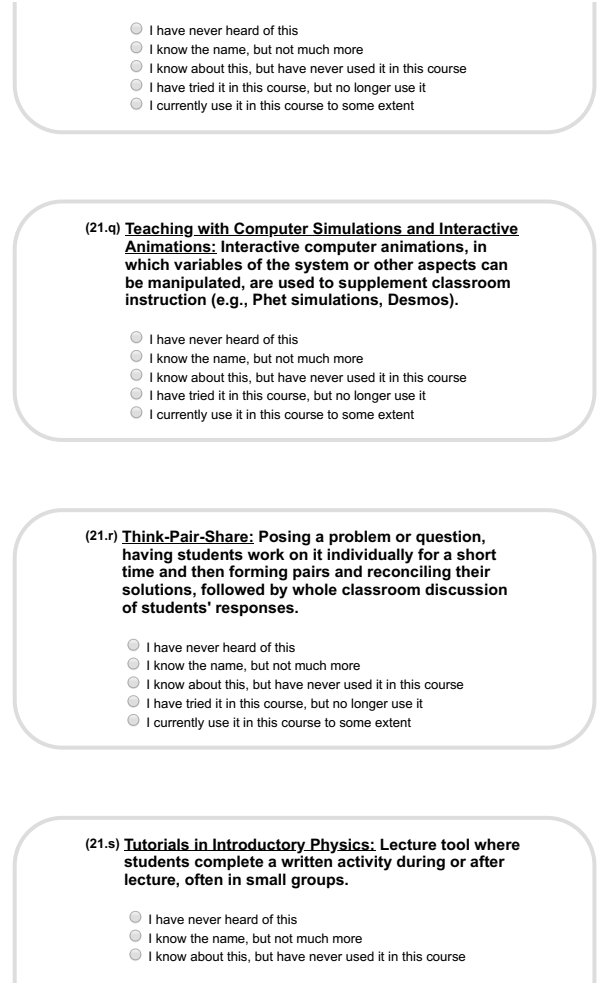


**Professional development experiences**


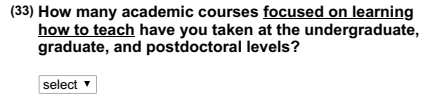


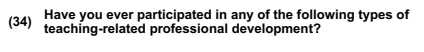


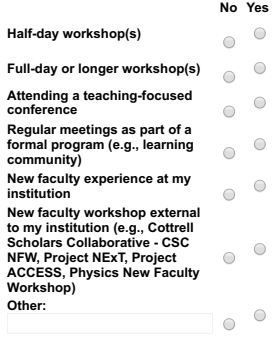


**Academic Rank**


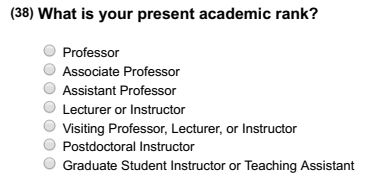


**Tenure Status**


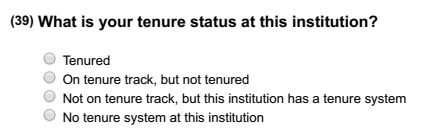


**Distribution of position**


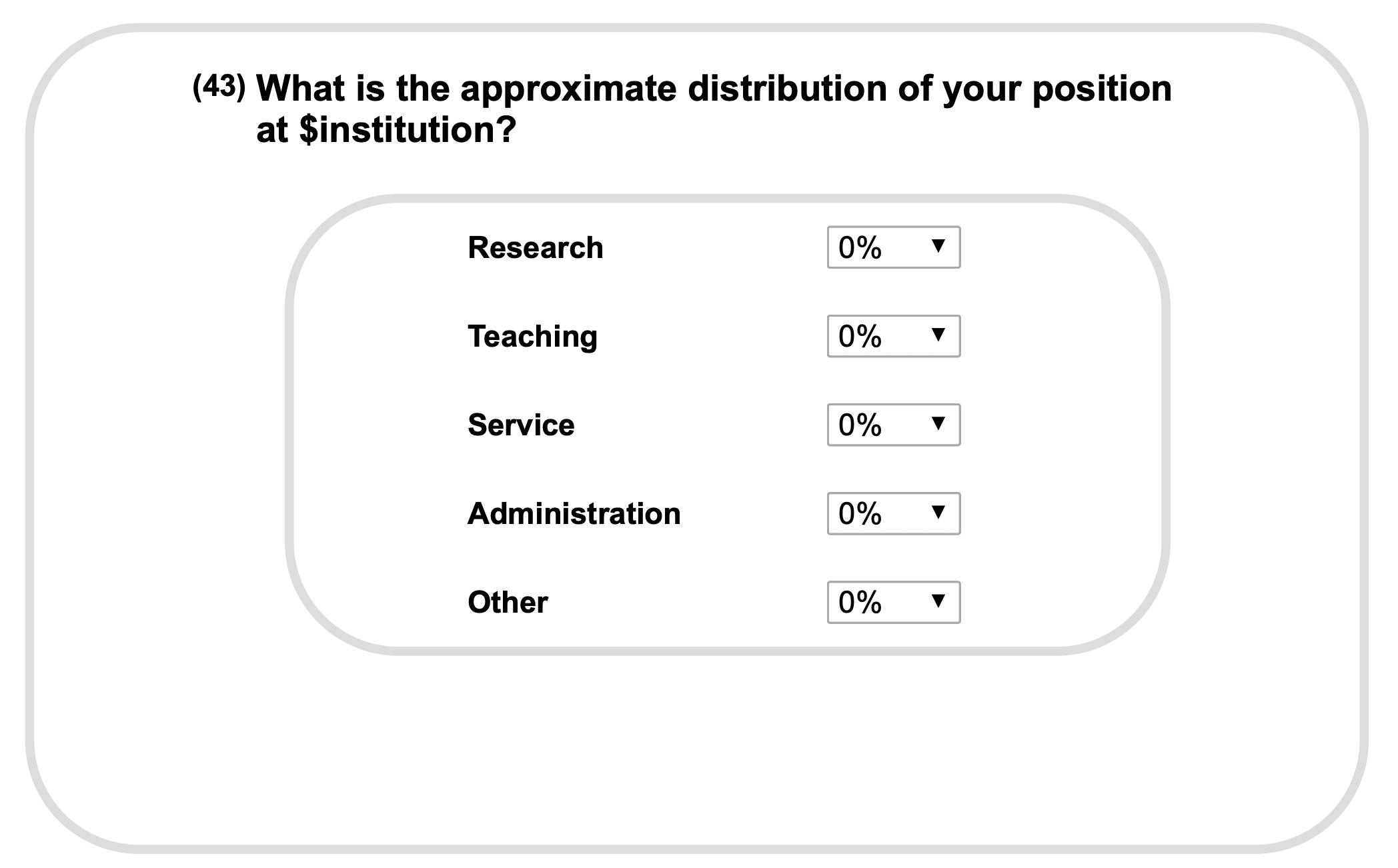


**Teaching Load**


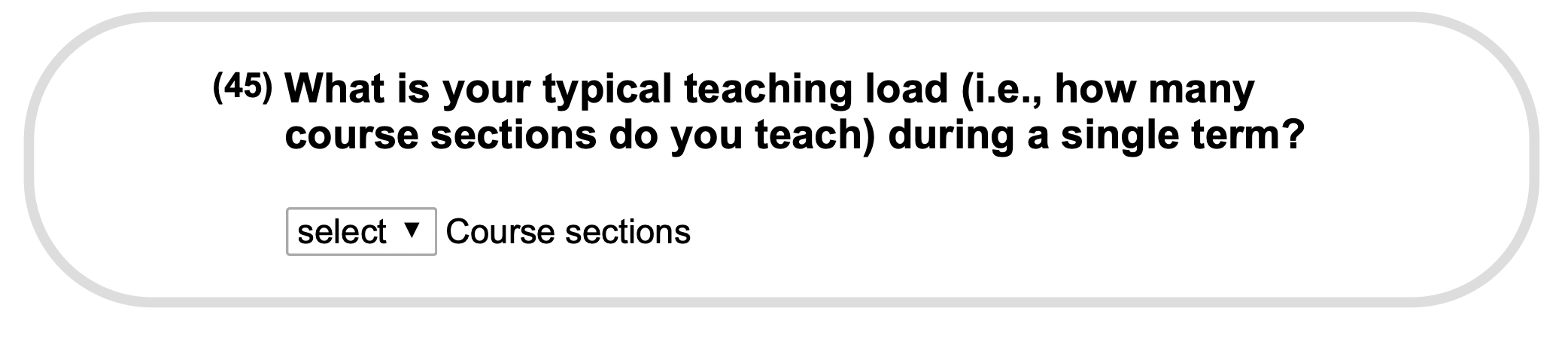


**Teaching Experiences**


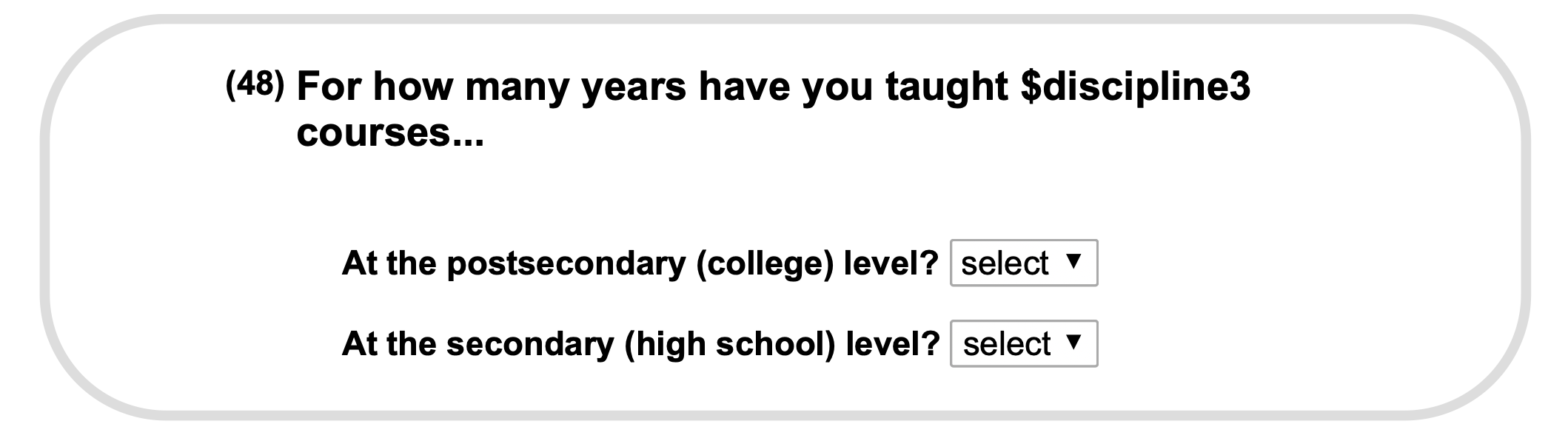


**Demographics**


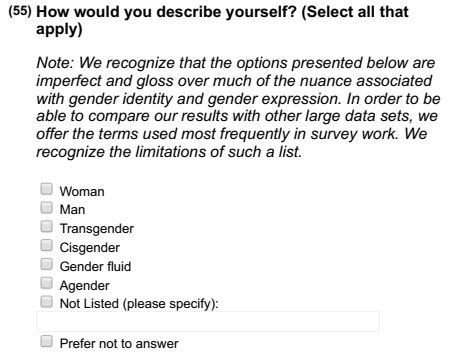


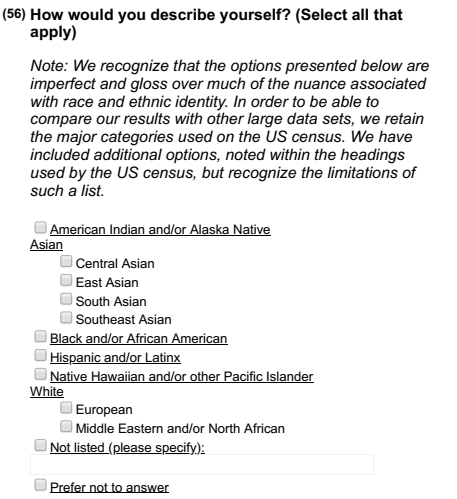


Table S1: Gender by Tenure Status by Institution Type

| Institution Type | Percentage of Total Participants within each Tenure Status in each Institution Type (%) | | | | | | | |
| --- | --- | --- | --- | --- | --- | --- | --- | --- |
|  | Tenured | | On tenure track | | Not on a tenure track | | No tenure system | |
|  | Women | Men | Women | Men | Women | Men | Women | Men |
| A.A. | 46.3 | 53.7 | 47.6 | 52.4 | 38.1 | 61.9 | 53.6 | 46.4 |
| B.A./B.S. | 35.3 | 64.7 | 45.3 | 54.7 | 75.0 | 25.0 |  |  |
| M.A./M.S. | 41.9 | 58.1 | 40.5 | 59.5 | 45.5 | 54.5 |  |  |
| Ph.D. | 18.5 | 81.5 | 40.0 | 60.0 | 53.4 | 46.6 |  |  |

Table S2: Race/Ethnicity by Tenure Status by Institution Type

| Institution Type | Percentage of Total Participants within each Tenure Status in each Institution Type (%) | | | | | | | |
| --- | --- | --- | --- | --- | --- | --- | --- | --- |
|  | Tenured | | On tenure track | | Not on a tenure track | | No tenure system | |
|  | European | Non-European | European | Non-European | European | Non-European | European | Non-European |
| A.A. | 77.9 | 22.1 | 63.6 | 36.4 | 72.1 | 27.9 | 91.4 | 8.6 |
| B.A./B.S. | 91.2 | 8.8 | 84.1 | 15.9 | 76.2 | 23.8 |  |  |
| M.A./M.S. | 79.3 | 20.7 | 56.4 | 43.6 | 76.7 | 23.3 |  |  |
| Ph.D. | 79.2 | 20.8 | 88.0 | 12.0 | 81.9 | 18.1 |  |  |

Table S3: Instructors’ Highest Degree by Institution Type

| Degree level | Percentage of Total Participants in each Institution Type (%) | | | |
| --- | --- | --- | --- | --- |
|  | A.A. | B.A./B.S. | M.A./M.S. | Ph.D. |
|  | (n=343) | (n=342) | (n=151) | (n=247) |
| Bachelor's level degree | 1.5 | 0.0 | 0.0 | 0.8 |
| Master's level degree | 32.4 | 2.6 | 7.9 | 5.7 |
| Doctoral level degree | 66.2 | 97.4 | 92.1 | 93.5 |

Table S4: Teaching Experiences at Postsecondary Level by Institution Type

| Years of Teaching | Percentage of Total Participants in each Institution Type (%) | | | |
| --- | --- | --- | --- | --- |
|  | A.A. | B.A./B.S. | M.A./M.S. | Ph.D. |
|  | (n=342) | (n=340) | (n=149) | (n=245) |
| 1-4 | 10.5 | 15.6 | 19.5 | 14.7 |
| 5-9 | 19.6 | 15.3 | 26.8 | 17.1 |
| 10-14 | 21.6 | 18.2 | 14.8 | 16.7 |
| 15 and 15+ | 48.2 | 50.9 | 38.9 | 51.4 |

Table S5. Number of Courses Taught Each Semester by Institution Type

| Institution Type | n | Mean | SE |
| --- | --- | --- | --- |
| A.A. | 337 | 3.1 | 0.1 |
| B.A./B.S. | 340 | 3.2 | 0.0 |
| M.A./M.S. | 150 | 2.9 | 0.1 |
| Ph.D. | 246 | 1.9 | 0.1 |

Table S6. Enrollment in Class by Institution Type

| Institution Type | n | Mean | SE |
| --- | --- | --- | --- |
| A.A. | 415 | 30.4 | 0.8 |
| B.A./B.S. | 379 | 47.5 | 1.7 |
| M.A./M.S. | 167 | 79.1 | 4.1 |
| Ph.D. | 271 | 193.9 | 8.2 |

Table S7. Distributions of Decision Makers on Content and Topic Coverage, Textbook, Exams, and Instructional Methods

|  | Percentage of Total Participants in each Institution Type (%) | | |
| --- | --- | --- | --- |
|  | Myself | Myself and others | One or more other people |
| Instructional Methods | 85.3 | 14.0 | 0.7 |
| Exams | 83.2 | 15.6 | 1.2 |
| Content and Topic Coverage | 19.6 | 68.8 | 11.6 |
| Textbook | 18.0 | 64.4 | 17.6 |

Table S8. Distributions of Decision Makers on Instructional Methods by Institution Type

|  | Percentage of Total Participants in each Institution Type (%) | | |
| --- | --- | --- | --- |
|  | Myself | Myself and others | One or more other people |
| A.A. | 89.4 | 10.2 | 0.5 |
| B.A./B.S. | 88.3 | 11.7 | - |
| M.A./M.S. | 80.8 | 18.6 | 0.6 |
| Ph.D. | 77.8 | 20.4 | 1.9 |

Table S9. Distributions of Decision Makers on Exam by Institution Type

|  | Percentage of Total Participants in each Institution Type (%) | | |
| --- | --- | --- | --- |
|  | Myself | Myself and others | One or more other people |
| A.A. | 92.0 | 7.0 | 1.0 |
| B.A./B.S. | 84.6 | 15.1 | 0.3 |
| M.A./M.S. | 81.3 | 16.3 | 2.4 |
| Ph.D. | 68.9 | 28.9 | 2.2 |

Table S10. Distributions of Decision Makers on Content by Institution Type

|  | Percentage of Total Participants in each Institution Type (%) | | |
| --- | --- | --- | --- |
|  | Myself | Myself and others | One or more other people |
| A.A. | 27.1 | 55.8 | 17.1 |
| B.A./B.S. | 17.0 | 79.1 | 4.0 |
| M.A./M.S. | 11.5 | 71.1 | 17.5 |
| Ph.D. | 16.7 | 73.0 | 10.4 |

Table S11. Distributions of Decision Makers on Textbook by Institution Type

|  | Percentage of Total Participants in each Institution Type (%) | | |
| --- | --- | --- | --- |
|  | Myself | Myself and others | One or more other people |
| A.A. | 28.9 | 48.4 | 22.7 |
| B.A./B.S. | 13.6 | 77.1 | 9.3 |
| M.A./M.S. | 10.2 | 69.3 | 20.5 |
| Ph.D. | 12.3 | 68.0 | 19.7 |

Table S12. Distributions of RBIS Knowers and Users by Institution

|  | Percentage of Total Participants within each Tenure Status (%) | |
| --- | --- | --- |
|  | Knowers | Knowers and Users |
| A.A. | 73.1 | 43.6 |
| B.A./B.S. | 85.8 | 55.4 |
| M.A./M.S. | 80.3 | 53.5 |
| Ph.D. | 79.4 | 53.3 |

Table S13. Distributions of RBIS Knowers and Users by Tenure Status at A.A. Institutions

|  | Percentage of Total Participants within each Tenure Status (%) | |
| --- | --- | --- |
|  | Knowers | Knowers and Users |
| No tenure system | 66.2 | 30.4 |
| Not on tenure-track | 45.3 | 29.0 |
| Tenure-track | 79.1 | 50.0 |
| Tenured | 80.0 | 46.0 |

Table S14. Distributions of RBIS Knowers and Users by Tenure Status at B.A./B.S. Institutions

|  | Percentage of Total Participants within each Tenure Status (%) | |
| --- | --- | --- |
|  | Knowers | Knowers and Users |
| Not on tenure-track | 75.0 | 52.5 |
| Tenure-track | 84.4 | 59.4 |
| Tenured | 87.1 | 52.2 |

Table S15. Distributions of RBIS Knowers and Users by Tenure Status at M.A./M.S. Institutions

|  | Percentage of Total Participants within each Tenure Status (%) | |
| --- | --- | --- |
|  | Knowers | Knowers and Users |
| Not on tenure-track | 76.1 | 53.5 |
| Tenure-track | 81.1 | 55.6 |
| Tenured | 78.3 | 45.8 |

Table S16. Distributions of RBIS Knowers and Users by Tenure Status at Ph.D. Institutions

|  | Percentage of Total Participants within each Tenure Status (%) | |
| --- | --- | --- |
|  | Knowers | Knowers and Users |
| Not on tenure-track | 87.8 | 64.4 |
| Tenure-track | 76.0 | 68.0 |
| Tenured | 68.2 | 33.0 |

Table S17. Distribution of Instructors in terms of their Knowledge and Usage of Specific RBIS

|  | Percentage of Total Participants (%) | | | | |
| --- | --- | --- | --- | --- | --- |
|  | I have never heard of this | I know the name, but not much more | I know about this, but have never used it in my course | I have tried it in this course, but no longer use it | I currently use it in this course to some extent |
| Formal Small Group Work | 7.8 | 6.0 | 33.5 | 10.0 | 42.7 |
| Flipped Classroom | 4.0 | 2.6 | 54.2 | 10.2 | 29.1 |
| Think-Pair-Share | 17.7 | 8.8 | 28.9 | 6.7 | 37.9 |
| Peer-Led Team Learning (PLTL) | 12.4 | 8.9 | 39.1 | 8.0 | 31.5 |
| Teaching with Computer Simulations and Interactive Animations | 14.6 | 11.2 | 30.3 | 6.0 | 37.8 |
| Peer Instruction | 14.1 | 12.2 | 34.5 | 7.2 | 32.1 |
| Process Oriented Guided Inquiry Learning (POGIL) | 12.8 | 8.0 | 49.5 | 10.5 | 19.1 |
| Interactive Lecture Demonstrations | 19.9 | 9.4 | 35.5 | 7.0 | 28.1 |
| Concept Maps | 16.5 | 10.6 | 44.8 | 8.1 | 20.0 |
| Just-in-Time Teaching | 23.5 | 11.6 | 42.0 | 6.6 | 16.4 |
| Peer-Reviewed Scientific Writing | 22.1 | 12.4 | 53.3 | 5.8 | 6.5 |
| Concept Inventories | 37.0 | 13.4 | 24.3 | 4.7 | 20.7 |
| Studio/SCALE-UP | 47.8 | 9.5 | 27.1 | 2.1 | 13.5 |
| Chemical Thinking | 60.4 | 13.7 | 8.7 | 1.2 | 15.9 |
| Chemistry, Life, the Universe, and Everything (CLUE) | 57.5 | 13.3 | 18.8 | 0.6 | 9.8 |

Table S18: Descriptive Statistics of Percentage of Time Spent on Four Class Activities by Institution Type

| Institution Type | Percent of Time (%) | | | |
| --- | --- | --- | --- | --- |
|  | Participating in whole class discussions | Working individually | Working in small groups | Listening to the instructor lecture or solve problems |
| A.A. | 13.9 ± 3.4 | 13.3± 3.2 | 17.9± 4.2 | 55.0± 5.1 |
| B.A./B.S. | 9.8± 3.2 | 11.1± 2.8 | 21.5± 4.7 | 57.6± 5.5 |
| M.A./M.S. | 9.5± 3.8 | 11.2± 3.4 | 18.6± 4.9 | 60.7± 5.7 |
| Ph.D. | 8.8± 3.3 | 10.3± 3.1 | 14.8± 4.3 | 66.1± 5.2 |

Table S19: Statistics of Comparison Analysis on Percentage of Time Spent on Four Class Activities by Tenure Status by Institution

| Institution Type | Tenured | On tenure track | Not on tenure track | No tenure system | F value | p-value | $\eta^{2}$ |
| --- | --- | --- | --- | --- | --- | --- | --- |
| A.A. | 53.9 ± 2.0 | 53.3 ± 3.5 | 57.2 ± 2.0 | 58.5 ± 2.8 | 0.832 | 0.477 | 0.008 |
| B.A./B.S. | 58.4 ± 1.8 | 58.6 ± 2.6 | 53.1 ± 4.5 | - | 0.761 | 0.468 | 0.005 |
| M.A./M.S. | 63.9 ± 3.1 | 56.4 ± 4.0 | 61.1 ± 4.0 | - | 0.999 | 0.371 | 0.014 |
| Ph.D. | 69.5 ± 2.3 | 73.0 ± 3.4 | 58.4 ± 2.4 | - | 7.420 | 0.001 | 0.062 |

Table S20: Distribution of Academic Position by Institution Type

|  | AA | BA/BS | MA/MS | PhD |
| --- | --- | --- | --- | --- |
| Research | 2.0 ± 0.3 | 18.4 ± 0.7 | 20.8 ± 1.4 | 25.8 ± 1.5 |
| Teaching | 82.9 ± 1.0 | 63.4 ± 0.9 | 60.5 ± 2.0 | 54.0 ± 1.7 |
| Service | 8.4 ± 0.5 | 13.3 ± 0.4 | 12.2 ± 0.7 | 12.4 ± 0.6 |

**Figure S1: Distribution of Instructors by Number of Professional Development Types Attend in their Teaching Positions**


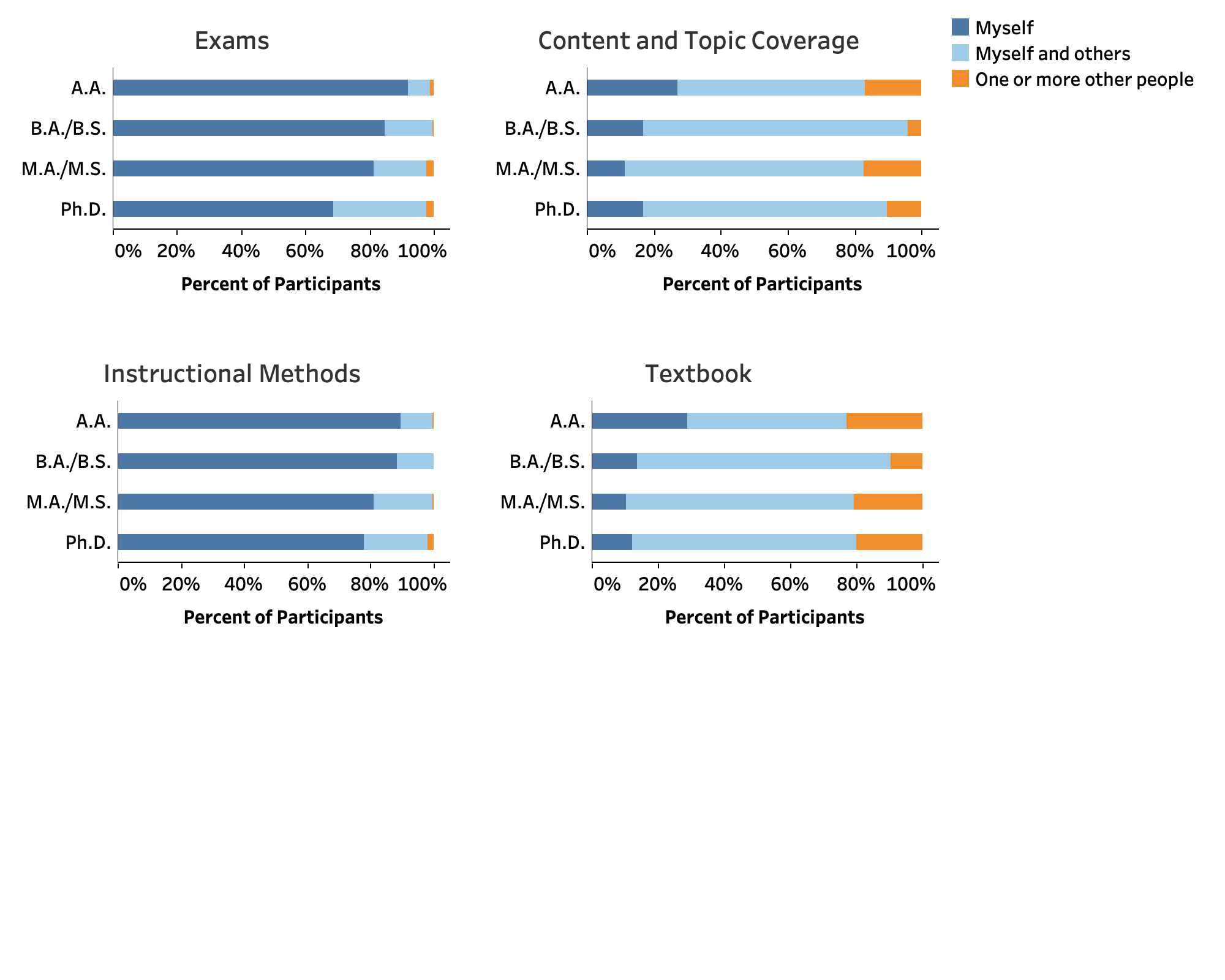


**Figure S2: Distributions of Decision Makers for Four Course Components by Institution Type (A.A.: n=414, B.A./B.S.: n=377, M.A./M.S.: n =167, Ph.D.: n=270)**


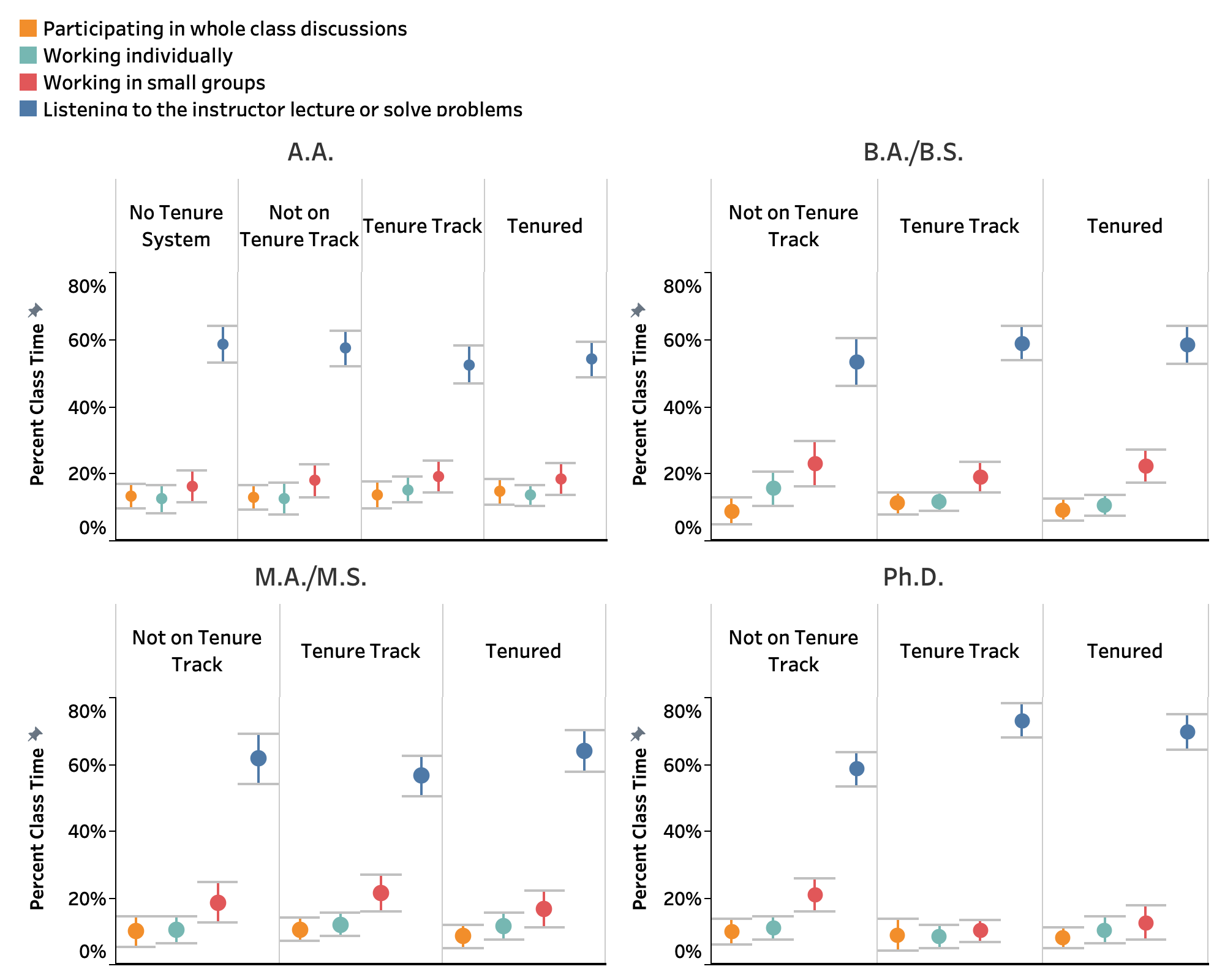


**Figure S3: Percentage of Time Spent on Four Class Activities by Tenure Status by Institution**
